# Balloon Dilatation in the Management of Congenital Obstructive Lesions of the Heart: Review of Author’s Experiences and Observations—Part I

**DOI:** 10.3390/jcdd10060227

**Published:** 2023-05-23

**Authors:** P. Syamasundar Rao

**Affiliations:** Children’s Heart Institute, University of Texas-Houston McGovern Medical School, Children’s Memorial Hermann Hospital, Houston, TX 77030, USA; p.syamasundar.rao@uth.tmc.edu or srao.patnana@yahoo.com; Tel./Fax: +1-713-500-5738

**Keywords:** pulmonary stenosis, aortic stenosis, aortic coarctation, balloon valvuloplasy, balloon angioplasty, long-term results, pulmonary insufficiency, aortic insufficiency, aortic aneurysm

## Abstract

Balloon dilatation techniques became available to treat congenital obstructive lesions of the heart in the early/mid-1980s. The purpose of this review is to present the author’s experiences and observations on the techniques and outcomes of balloon dilatation of pulmonary stenosis (PS), aortic stenosis (AS) and aortic coarctation (AC), both native and postsurgical re-coarctations. Balloon dilatation resulted in a reduction of peak pressure gradient across the obstructive lesion at the time of the procedure as well as at short-term and long-term follow-ups. Complications such as recurrence of stenosis, valvar insufficiency (for PS and AS cases) and aneurysm formation (for AC cases) have been reported, but infrequently. It was recommended that strategies be developed to prevent the reported complications.

## 1. Introduction

In 1964, Dotter and Judkins [1] dilatated stenotic lesions of peripheral arteries by advancing guide wires across the obstructive lesions followed by passing of gradually increasing sizes of dilating catheters; they reported good results both immediately after the procedure and during follow-up [1]. Slightly more than a decade later, Grüntzig and his colleagues utilized Dotter’s principle and developed double-lumen catheters with balloons [2] and employed these balloon catheters to dilate stenotic lesions of the iliac, femoral, popliteal [2], renal [3], and coronary [4,5,6] arteries. Pediatric applications of these techniques followed by using similar balloon catheters to enlarge aortic coarctation (AC) [7,8,9], pulmonary stenosis (PS) [10], and aortic stenosis (AS) [11,12] in the early/mid-1980s. The author applied these balloon dilatation techniques in his clinical practice to address congenital stenotic lesions of the heart and published his experiences and results in local, national, and international journals [13,14,15,16,17,18,19,20,21,22,23,24,25,26,27,28,29,30,31], books [32,33] and book chapters [34,35,36,37,38,39]. Subsequently, short-term [40,41,42,43,44,45,46] and long-term [47,48,49,50,51] outcomes were examined. The purpose of this review is to present the author’s experiences and observations on the role of balloon dilatation techniques in the management of congenital obstructive lesions of the heart. While balloon dilatation techniques are useful in treating many other obstructive lesions of the heart and vascular structures as listed in [52,53,54,55,56,57,58,59], this presentation is limited to PS, AS, and AC because of limitations of space.

## 2. Indications for Balloon Dilatation Procedures

It is generally thought that the indications for transcatheter interventions should be the same as those used for surgical therapy [60,61,62,63,64,65]. Indications for balloon dilatation for each of the lesions (PS, AS, and AC) will be reviewed separately.

### 2.1. Pulmonary Stenosis

The author recommended that a peak-to-peak systolic pressure gradient of 50 mmHg or more across the pulmonary valve be used as an indication for balloon pulmonary valvuloplasty (BPV) [60,62,66]. However, the American Heart Association’s Committee on Guidelines for Intervention in Pediatric Cardiac Disease [67] suggested a peak gradient of 40 mmHg across the pulmonary valve or a right ventricular peak systolic pressure of 50 mmHg as indications for BPV. Due to the concern for adverse effects of this lowered criteria for selection of patients for BPV, the author examined the results of studies that used less than 50 mmHg gradients for BPV [60]; these data revealed the following: 1. Decrease in right ventricular (RV) peak systolic pressures from 60 ± 9 to 43 ± 11 mmHg (*p* < 0.01) and of pulmonary valve peak-to-peak gradients from 38 ± 6 to 20 ± 11 mmHg (*p* < 0.01) occurred immediately after BPV and 2. Residual RV peak systolic pressures of 45 ± 12 mmHg and residual pulmonary valve gradients of 24 ± 12 mmHg were found at follow-up. This analysis concluded that there was only a marginal reduction in RV systolic pressure (60 vs. 45 mmHg) at follow-up. A review of natural history study data [68] indicated that trivial and mild PS (gradients < 50 mmHg) remained mild at follow-up. The availability of Doppler studies which are accurate in quantitating the degree of pulmonary valve obstruction [69], would easily identify if the degree of PS increases. Furthermore, accounts of significant pulmonary insufficiency at late follow-up after BPV [50,70,71,72], some requiring pulmonary valve replacement, give additional support to the idea of non-intervention in subjects with mild PS. The above-referred analysis and editorial [60] were published more than three decades ago and the author continues to support these concepts [50,66,73,74,75].

The above gradient criteria are based on pressure measurements secured during cardiac catheterization performed under conscious sedation. However, currently, most catheter interventional procedures in children are accomplished with general anesthesia; it is generally agreed that the valvar gradients are lower in subjects under general anesthesia than under conscious sedation protocol. Consequently, the criteria referred to above are not appropriate. Therefore, the pre-catheterization gradients derived by Doppler (peak instantaneous gradients) are used for arriving at a decision on the necessity for BPV. When using Doppler, the impact of the pressure recovery phenomenon [76,77] should be considered and appropriate correction to correct for pressure recovery should be employed.

Recurrent stenosis following previous surgical pulmonary valvotomy or BPV is also an indication for balloon valvuloplasty, subject to meeting pressure gradient criteria.

Pulmonary valve dysplasia is commonly deemed a relative contraindication for BPV. The author’s personal experience [78] and that reported by others [79] suggest that BPV is the initial management option. However, it should be known that good relief of obstruction will occur only if there is pulmonary valve commissural fusion. Based on our experience [78], balloon/annulus (B/A) ratios between 1.4 and 1.5 are likely to be successful in patients with dysplastic pulmonary valves [78].

It has been suggested by some cardiologists not to intervene in adult subjects with moderate to severe pulmonary stenosis if they do not have symptoms [80]. However, we recommend BPV to alleviate the obstruction in adult patients with moderate to severe PS, irrespective of the symptoms, because of inadequate response to exercise [81] and the possible onset of myocardial fibrosis [73,74,75,82].

### 2.2. Aortic Stenosis

Indications for balloon aortic valvuloplasty (BAV) are aortic valve peak gradient ≥ 50 mmHg with either symptoms or ST-T wave changes in the electrocardiogram (ECG) indicative of myocardial perfusion abnormality or a peak gradient more than 70 mmHg irrespective of the symptoms or ECG changes [31,48,63,83,84]. The considerations for using Doppler gradients instead of catheter gradients are the same as those described in the “Pulmonary Stenosis” section; correction for pressure recovery [76,77] should be applied.

Restenosis following previous surgical aortic valve surgery or BAV is also an indication for balloon valvuloplasty subject to meeting the pressure gradient criteria. Moderate to severe regurgitation of the aortic valve is a general contraindication for BAV secondary to fear of further increasing aortic regurgitation.

Elderly patients with calcific aortic stenosis are no longer candidates for BAV. While the initial results suggested that the BAV is beneficial in this subset of patients [63,85,86,87,88], it was subsequently determined that the relief of aortic valve obstruction is transient and not long-lasting [89,90]. Currently, elderly patients with calcific AS are addressed with transcatheter aortic valve replacement (TAVR) [91,92,93]. TAVR is not addressed in this review.

### 2.3. Aortic Coarctation, Native

The indications for balloon angioplasty (BA) of AC are significant hypertension and/or congestive heart failure (CHF) along with a peak-to-peak systolic pressure gradient of 20 mmHg or more across AC [16,19,27,39,43,46,94].

Infants (beyond one month of age), children, adolescents, and adults who do not have symptoms should have relief of the obstruction across AC on an elective basis. Simple observation or treatment with antihypertensive medicines without relieving the obstruction across AC is not prudent because even with effective treatment later, the patients may end up with residual hypertension [95,96]. Relief of the AC should be undertaken before one year of age to avoid systemic hypertension later in life [96].

While the author believes that BA of AC in neonates is feasible and effective [97,98,99,100,101], others [102] recommend surgical therapy of AC in neonates. The latter is largely due to the high rate of recurrent obstruction [97,98,102]. Nevertheless, the author advocates BA in special situations [103] namely, newborn babies with a shock-like presentation with severe cardiorespiratory distress [104], severe myocardial failure due to “hypertensive cardiomyopathy” because of AC [105], previous cerebral hemorrhage [103], and liver dysfunction secondary to biliary atresia [103]. Development of aneurysms during follow-up detected in some studies [106] is of concern for BA.

While BA is feasible in discrete and short-segment ACs, long-segment ACs are unlikely to be relieved by BA and these patients are candidates for implantation of stents [94,107,108,109].

### 2.4. Aortic Coarctation, Postsurgical

While there is no general agreement about whether surgery or BA is favored for native AC, there is a broad agreement among cardiologists and surgeons that BA is the treatment of choice for postsurgical aortic re-coarctations [27,45,46,49,61,65,110,111]. This is apparently due to substantial mortality and morbidity seen with repeat surgical intervention to address postsurgical re-coarctation [49,112,113,114]. Indications for BA are the same as those utilized for native AC: significant hypertension and/or CHF with a peak systolic gradient of 20 mmHg across the re-coarctation site [45,49,65]. Patients with long-segment re-coarctations are candidates for stent deployment [94,107,108,109].

**Figure 1:**
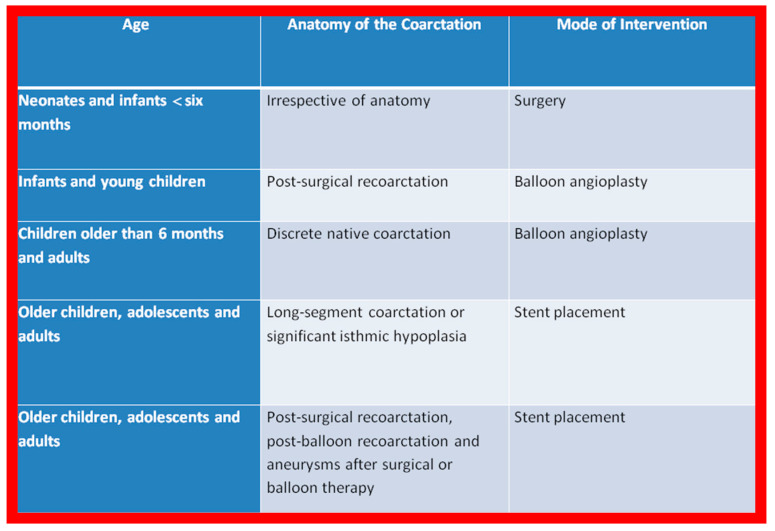
The author’s recommendations specify the type of therapy depending upon the patient’s age and type of coarctation. Reproduced from Reference [94].

Based on these observations and a review of the applicable literature, the author’s recommendations are tabulated in Figure 1.

## 3. Techniques of Balloon Dilatation

The techniques of balloon valvuloplasty for PS [16,62,66,73,74,75,115] and AS [83,84,116] and BA for AC [16,44,45,49,64,65,94] were detailed elsewhere and these will be briefly reviewed in this section.

### 3.1. Pulmonary Stenosis

**Figure 2:**
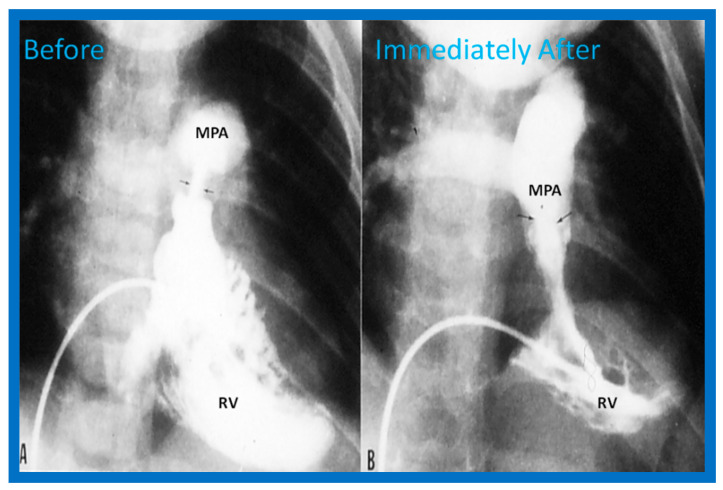
Selected frames from right ventricular (RV) cine-angiograms in a sitting-up (15° left anterior oblique and 35° cranial) view before (**A**) and immediately after (**B**) balloon pulmonary valvuloplasty. Note the thin jet (arrows in (**A**)) prior to valvuloplasty which increased in width (arrows in (**B**)) after valvuloplasty. C, Catheter; MPA, main pulmonary artery. Reproduced from Reference [62].

**Figure 3:**
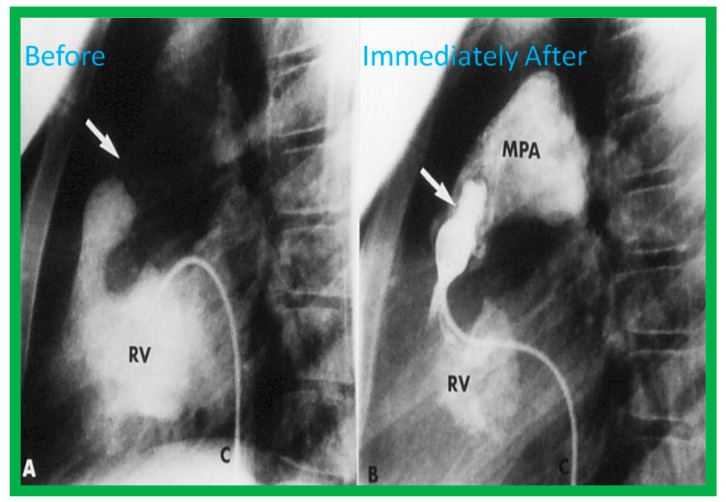
Selected frames from right ventricular (RV) cine-angiograms in lateral views before (**A**) and immediately after (**B**) balloon pulmonary valvuloplasty. Note the thin jet (barely seen) (arrow in (**A**)) prior to valvuloplasty which increased in width (arrow in (**B**)) after valvuloplasty. C, Catheter; MPA, main pulmonary artery. Reproduced from Reference [32].

Once the criteria for BPV are fulfilled, cardiac catheterization is performed. The femoral venous route is the most often used catheter entry site. In patients with obstructed femoral/iliac veins, and those who have infra-hepatic interruption of the inferior vena cava with azygos or hemiazygos continuation, the right jugular venous approach is used. After securing hemodynamic data, RV cine-angiogram in sitting-up and lateral projections is obtained (Figure 2A and Figure 3A).

**Figure 4:**
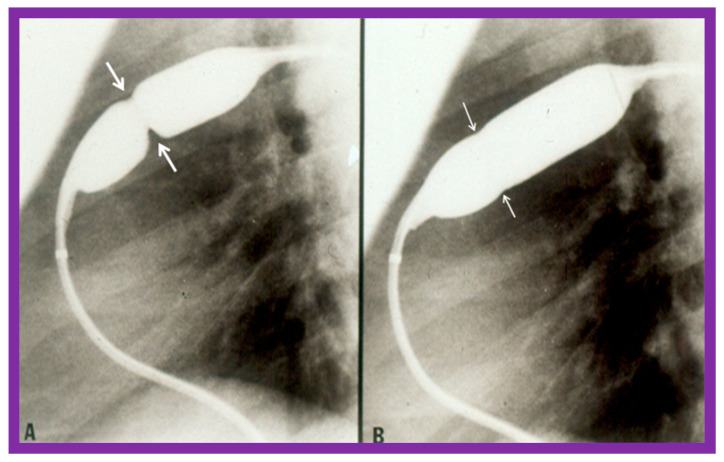
Balloon dilatation catheter placed across the pulmonary valve showing “waisting” of the balloon (**A**) in the early phases of inflation (thick arrows) which is almost completely abolished with further balloon inflation (**B**) (thin arrows). Modified from Reference [62].

**Figure 5:**
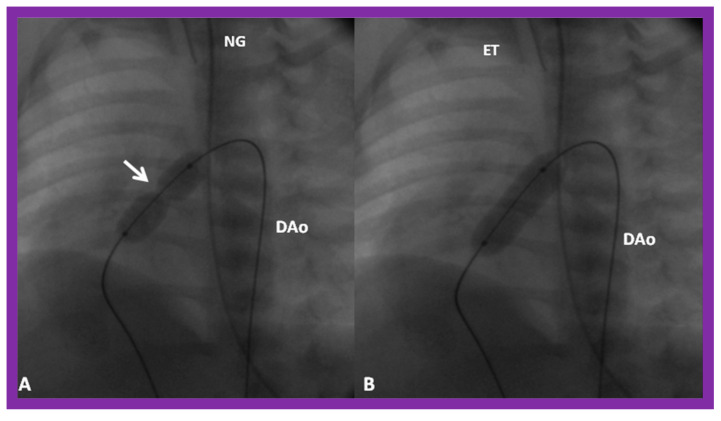
(**A**) A selected cine frame in 15° left anterior oblique with a 35° cranial angulated view demonstrates the position of a balloon angioplasty catheter across the stenotic pulmonary valve. Note the waisting of the balloon (arrow) during the early phase of balloon inflation. (**B**) The waist has been completely abolished on further inflation of the balloon. Note that the guide wire is passing through the ductus into the descending aorta (DAo). ET, endotracheal tube; NG, nasogastric tube. Reproduced from Reference [100].

Pulmonary valve annulus diameter is measured which is used for the selection of size of the balloon catheter used for BPV. At the present time, a balloon catheter that gives a balloon/annulus ratio of 1.2 to 1.25 is chosen for performing BPV [66,71,72]. The selected balloon catheter is positioned across the stenotic pulmonary valve over an extra-stiff, exchange-length guide wire, and the balloon is inflated with diluted contrast material until the waisting of the balloon is eliminated (Figure 4 and Figure 5).

**Figure 6:**
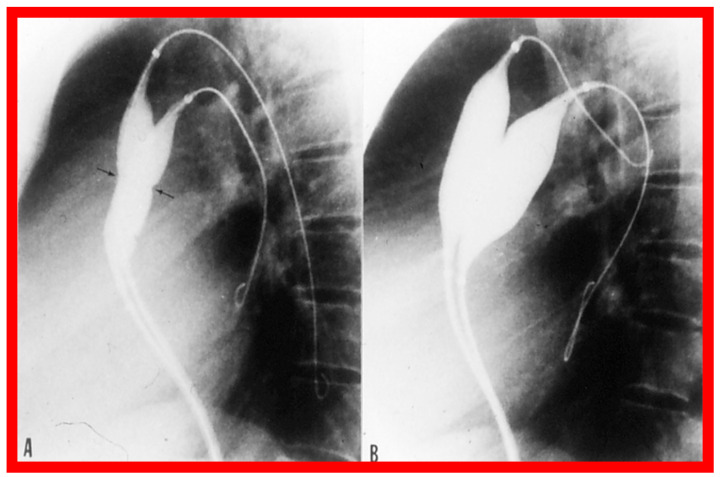
Selected cine-radiographic frames in lateral view, demonstrating two balloon catheters placed across the pulmonary valve, showing “waisting” of the balloons (arrows) during the initial phases of balloon inflation (**A**), which was completely abolished after the complete inflation of the balloons (**B**). Reproduced from Reference [62].

If the pulmonary valve annulus is too large to dilate with a single balloon, a double-balloon technique is used (Figure 6). The effective balloon size is calculated using the following formula [117]:

D_1_ + D_2_ + Π (D_1_/2 + D_2_/2)/Π

where D_1_ and D_2_ are the diameters of the balloons used.

This formula was further simplified to 0.82 (D_1_ + D_2_) by Narang et al. [118].

However, because of the availability of large-sized balloons at the present time, the need for using the double-balloon technique has diminished.

Following completion of the procedure, post-BPV hemodynamic data and RV cine-angiogram (Figure 2B and Figure 3B) are secured, and the catheters and sheaths are removed. Some cardiologists avoid repeating RV angiograms and instead use echocardiography to evaluate post-BPV status. For a more detailed description of the BPV procedure, the reader is referred to our recent reviews [75,115].

#### Pulmonary Stenosis in the Fetus

The description of BPV in the fetus was reviewed in detail elsewhere [115] and will not be discussed here because of the limitation of space.

### 3.2. Aortic Stenosis

**Figure 7:**
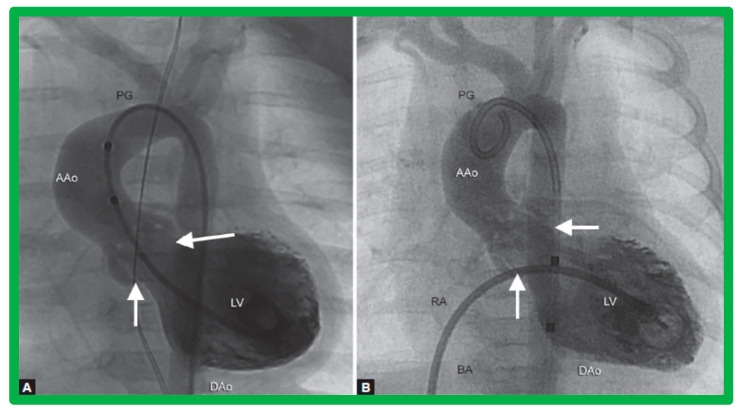
Selected cine frames from left ventricular (LV) cine-angiograms in posterior-anterior view in two neonates with severe aortic stenosis. (**A**) A pigtail (PG) catheter was introduced into the LV retrogradely; (**B**) A Berman angiographic (BA) catheter was advanced from the right atrium (RA), across a patent foramen ovale (not marked) into the left atrium (not marked) and from there into the LV. These angiograms demonstrate the aortic valve annulus (arrows in (**A**,**B**)). Note the domed and thickened aortic valve leaflets. Reproduced from Reference [127].

After fulfilling the criteria for BAV, cardiac catheterization is performed. The femoral arterial route is the most often used catheter entry. However, because of concern for femoral arterial injury in the neonate and young infant, other access sites, namely, subscapular [119], axillary [120], carotid [121], and umbilical [122] arterial, anterograde femoral venous [123,124], and anterograde umbilical venous [125,126] routes may be used for performing BAV. After obtaining hemodynamic information, aortic root and left ventricular cine-angiogram in the left anterior oblique and right anterior oblique projections are obtained (Figure 7).

**Figure 8:**
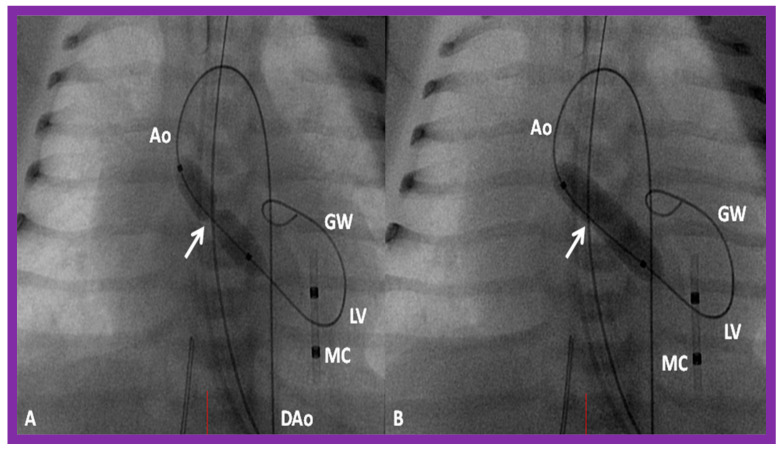
Selected cine frames in posterior–anterior projections illustrating a balloon dilatation catheter across the stenosed aortic valve. Waisting of the balloon (arrow) was seen during the early phases of inflation of the balloon (**A**) which was completely abolished on further inflation of the balloon (**B**). Ao, aorta; DAo, descending aorta; GW, guide wire; LV, left ventricle; MC, marker catheter. Reproduced from Reference [84].

The aortic valve annulus diameter is measured which is used for the selection of size of the balloon catheter used for BAV. At the present time, a balloon catheter that gives a balloon/annulus ratio of 0.8 to 1.0 is chosen for performing BAV [42,48,63]. The selected balloon catheter is positioned across the stenotic aortic valve over an extra-stiff, exchange-length guide wire, and the balloon is inflated with diluted contrast material until the waisting of the balloon is abolished (Figure 8). The considerations for using a double-balloon technique are like those stated in the “Pulmonary Stenosis” section.

Following the conclusion of the procedure, post-BAV hemodynamic data and left ventricular (LV) and aortic root cine-angiograms are obtained and the catheters and sheaths are removed. For a more detailed description of the BAV procedure, the reader is referred to our recent review [116].

#### Aortic Stenosis in the Fetus

The description of BAV in a fetus was detailed elsewhere [116] and will not be reviewed here because of the limitation of space.

### 3.3. Aortic Coarctation, Native

**Figure 9:**
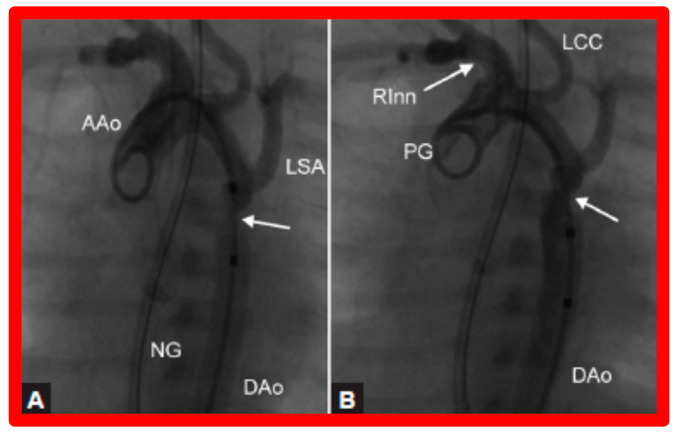
Selected cine frames from ascending aortic (AAo) cine-angiogram in 20° left anterior oblique projection demonstrating (**A**) narrowed coarcted aortic segment (arrow) prior to balloon angioplasty (**B**) which increased following balloon angioplasty. Note mild hypoplasia of the distal transverse aortic arch and isthmus. DAo, descending aorta; LCC, left common carotid artery; LSA, left subclavian artery; NG, nasogastric tube; PG, pigtail catheter; RInn, right innominate artery. Reproduced from Reference [94].

**Figure 10:**
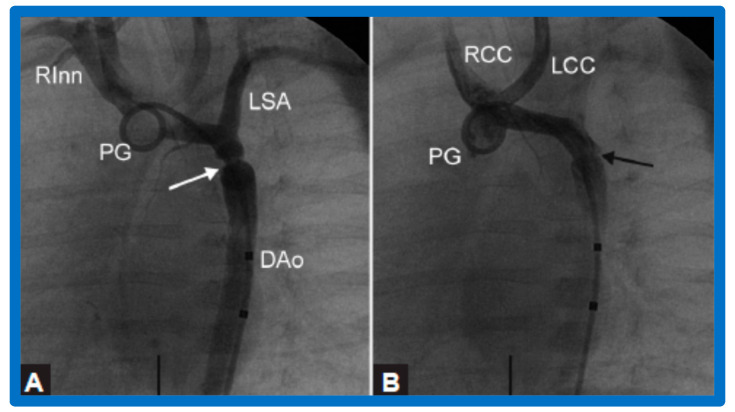
Selected aortic cine-angiographic frames in 20° left anterior oblique projection demonstrating (**A**) narrowed coarcted aortic segment (arrow) prior to balloon angioplasty (**B**) which increased following balloon angioplasty. DAo, descending aorta; LCC, left common carotid artery; LSA, left subclavian artery; PG, pigtail catheter; RCC, right common carotid artery; RInn, right innominate artery. Reproduced from Reference [94].

After fulfilling the criteria for BA of AC, cardiac catheterization is performed. The femoral arterial route is the most often used catheter entry site. However, because of concern for femoral arterial injury in the neonate, the umbilical arterial route [97,98,104,128] may be used for performing BA. A transvenous route may be used in patients with transposition of the great arteries, double outlet right ventricle, or large ventricular septal defect [129,130]. After obtaining hemodynamic information, including pressure pullback across the coarcted segment, an aortic arch cine-angiogram in the left anterior oblique and straight lateral projections is obtained (Figure 9A and Figure 10A).

**Figure 11:**
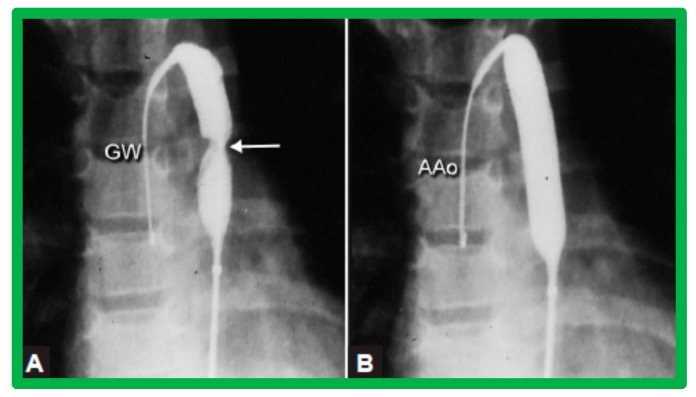
Selected cine-fluorographic frames in posteroanterior projection in a child demonstrating (**A**) an angioplasty balloon across the aortic coarctation with waisting (arrow) of the balloon (**B**) during the initial phases of balloon inflation; the waist has completely disappeared with further balloon inflation. The guidewire (GW) is positioned in the ascending aorta (AAo). Modified from Reference [64].

Various segments of the aorta including the diameter of the coarcted segment and of the descending aorta at the level of the diaphragm are measured; these are used for the selection of the size of the balloon catheter utilized for BA. The diameter of the balloon selected for BA is two or more times the diameter of the coarcted segment, but it should not be larger than the diameter of the descending aorta at the level of the diaphragm [16,64,130]. First, a balloon whose diameter is in-between the sizes of the aortic isthmus (or transverse aortic arch) and the descending aortic diameter at the level of the diaphragm is selected for BA. The chosen balloon catheter is positioned across the stenotic aortic segment over an extra-stiff, exchange-length guide wire, and the balloon is inflated with diluted contrast material until the waisting of the balloon is abolished (Figure 11). If no adequate relief of obstruction with gradients > 20 mmHg and no angiographic improvement has occurred, repeat BA with a balloon as large as the diameter of the descending aorta at the level of the diaphragm is undertaken [64,131,132,133].

Following the conclusion of the procedure, post-BA hemodynamic data and aortogram (Figure 9B and Figure 10B) are obtained and the catheters and sheaths are removed. For a more detailed description of the BA procedure, the reader is referred to our review [94].

### 3.4. Aortic Coarctation, Postsurgical

The technique of BA of postsurgical re-coarctation is the same as that described for native CA [45,65,111,132] and will not be repeated.

## 4. Immediate Results

The immediate results of the balloon procedures are assessed by hemodynamic and angiographic data secured fifteen minutes following the procedure and by echo-Doppler evaluation, usually performed on the morning after the procedure. Each of the lesions will be reviewed separately.

### 4.1. Pulmonary Stenosis

**Figure 12:**
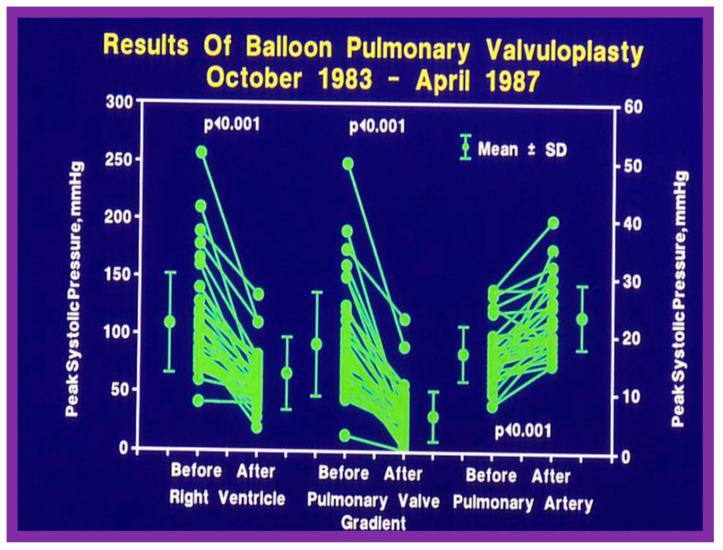
Line graph showing the immediate results of balloon pulmonary valvuloplasty. Note a significant (*p* < 0.001) decrease in the right ventricular peak systolic pressure (left panel) and the peak-to-peak systolic pressure gradient across the pulmonary valve (middle panel). Additionally shown is a slight but significant (*p* < 0.001) increase in the peak systolic pressure in the pulmonary artery (right panel). The mean ± standard deviation (SD) is shown. Reproduced from Reference [75].

**Figure 13:**
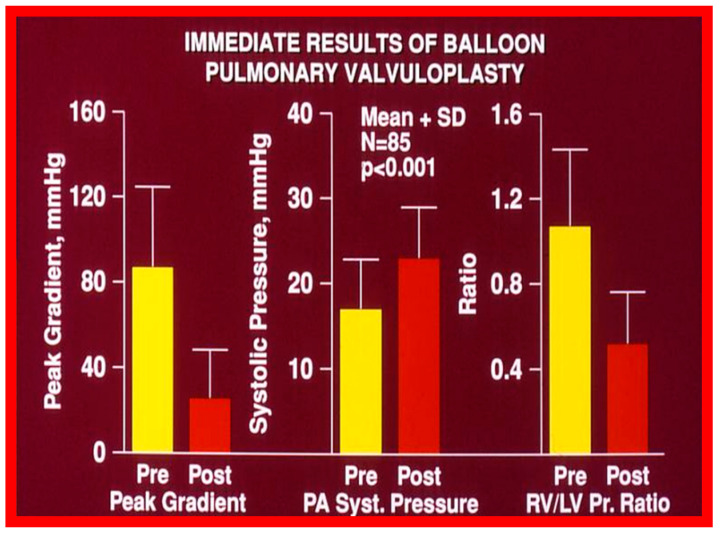
Bar graph showing the immediate results of balloon pulmonary valvuloplasty in 85 consecutive patients. Note a significant (*p* < 0.001) decrease in the peak-to-peak systolic pressure gradient across the pulmonary valve (left panel), a significant (*p* < 0.001) increase in the peak systolic (syst) pressure in the pulmonary artery (PA) (middle panel) and a significant (*p* < 0.001) decrease in the right ventricle (RV) to left ventricle (LV) systolic pressure (Pr.) ratio (right panel). The mean + standard deviation (SD) is shown. Pre, prior to balloon pulmonary valvuloplasty; Post, following balloon pulmonary valvuloplasty. Reproduced from Reference [136].

**Figure 14:**
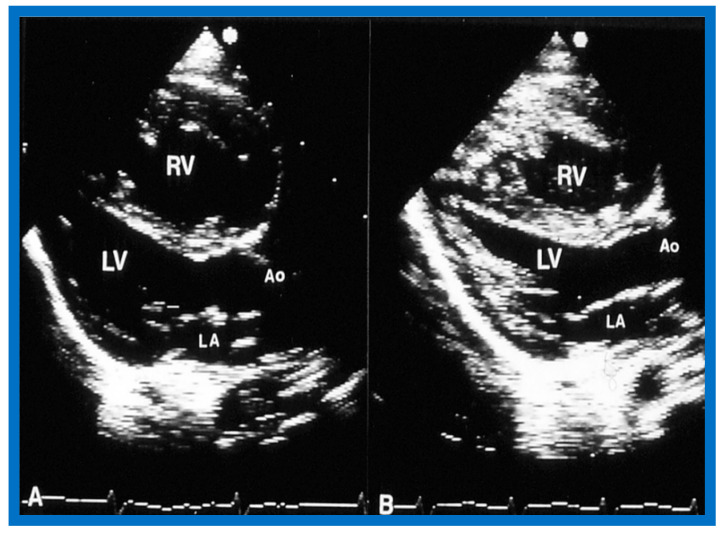
Selected video frames of two-dimensional images from parasternal long axis view prior to (**A**) and following (**B**) balloon pulmonary valvuloplasty, demonstrating a decrease in the size of the right ventricle (RV). Ao, aorta; LA, left atrium; LV, left ventricle. Reproduced from Reference [136].

**Figure 15:**
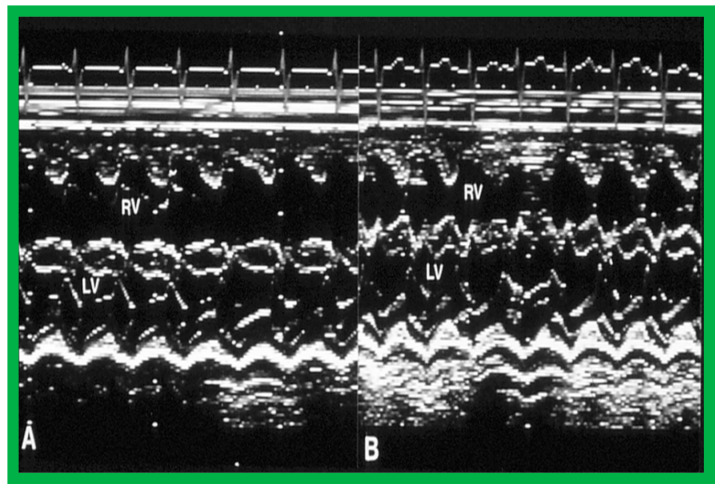
Selected m-mode tracings from parasternal long axis view prior to (**A**) and following (**B**) balloon pulmonary valvuloplasty, demonstrating a decrease in the size of the right ventricle (RV). LV, left ventricle. Reproduced from Reference [136].

**Figure 16:**
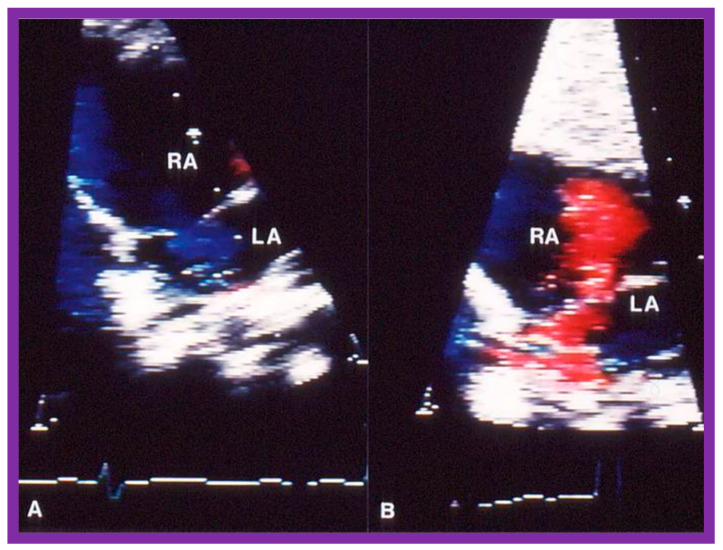
Selected video frames of the atrial septum, demonstrating a right-to-left shunt by color Doppler, across the patent foramen ovale prior to balloon pulmonary valvuloplasty (**A**) which has changed to a left-to-right shunt (**B**) 24 h later. LA, left atrium; RA, right atrium. Reproduced from Reference [75].

Immediate results of BPV were examined by the author in the mid-1980s [14,16]; subsequently, immediate results of a larger number of children [34,41,50,62] were published. Decrease in peak systolic pressure gradients across the pulmonary valve and RV peak systolic pressures occurred along with a minimal rise in pulmonary artery pressures (Figure 12 and Figure 13) after BPV without a change in the cardiac index. The jet width of contrast material across the pulmonary valve becomes wider (Figure 2B and Figure 3B). The size of the right ventricle decreased (Figure 14 and Figure 15). On post-BPV angiographic and echocardiographic examination, pulmonary valve leaflet excursion seemed freer with less doming. Right-to-left atrial shunt, if present prior to BPV, was reduced, eliminated, or reversed (Figure 16). Nevertheless, some patients with very severe PS developed obstruction at the RV infundibulum [134], some needing beta-blocker administration [134,135]. The topic associated with the development of infundibular stenosis will not be reviewed in this paper because of space limitations; the interested reader may find this information in other publications [134,135]. Surgical intervention was averted in most, if not all children. All patients except for newborns were sent home within 24 to 48 h after BPV [14,16,32,41,50,62]. The immediate results of BPV reported by other investigators are generally like those of ours and have been detailed elsewhere [25,50,62,75].

#### Pulmonary Stenosis in the Fetus

The description of the results of BPV in fetuses was detailed elsewhere [115] and will not be reviewed here because of the limitation of space.

### 4.2. Aortic Stenosis

**Figure 17:**
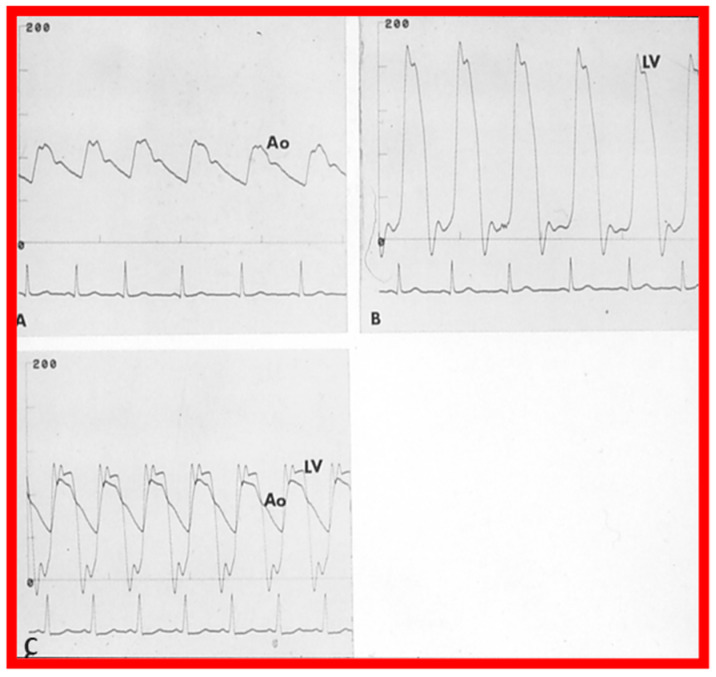
Aortic (Ao) and left ventricular (LV) pressure tracings prior to (**A**,**B**) and fifteen minutes following (**C**) balloon aortic valvuloplasty demonstrating almost complete abolition of the peak-to-peak pressure gradient across the aortic valve. Reproduced from Reference [63].

**Figure 18:**
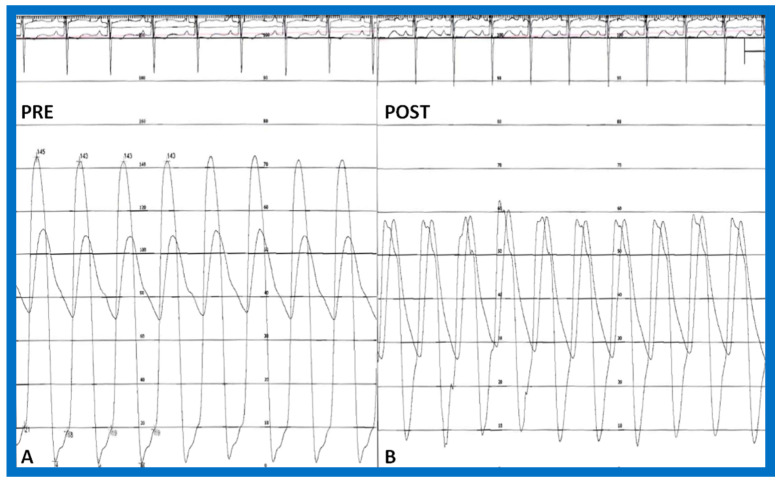
Simultaneous pressure recordings from the left ventricle and aorta prior to (PRE—(**A**)) and fifteen minutes following (POST—(**B**)) balloon aortic valvuloplasty demonstrating no residual gradient. There is a slight decrease in aortic diastolic pressure (**B**) suggesting aortic insufficiency. Reproduced from Reference [84].

**Figure 19:**
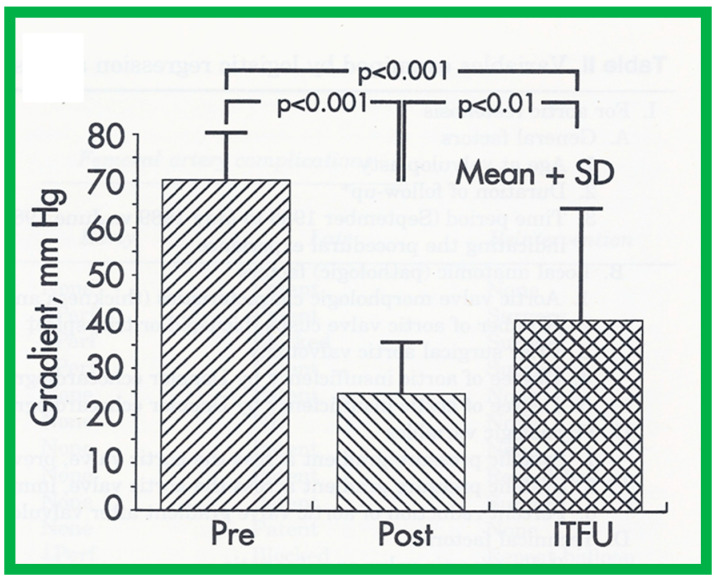
Bar graph demonstrating immediate and follow-up results after balloon aortic valvuloplasty. Note a significant (*p* < 0.001) decrease in peak-to-peak systolic pressure gradients across the aortic valve after balloon valvuloplasty (Pre, before vs. Post, immediately after). Gradient measured during repeat catheterization in 15 patients increased (*p* < 0.01) at intermediate-term follow-up (ITFU) of mean of 16 months. Reproduced from Reference [48].

**Figure 20:**
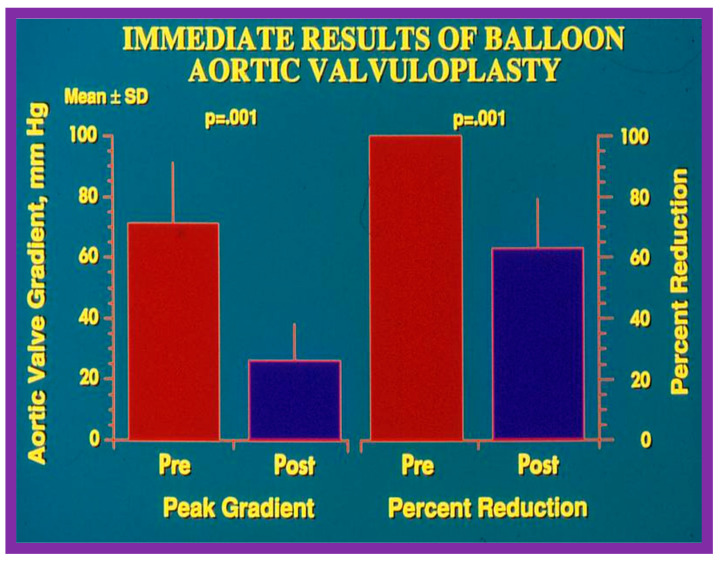
Bar graph illustrating immediate results of balloon aortic valvuloplasty for aortic valve stenosis. Significant (*p* = 0.001) decrease in the peak-to-peak systolic pressure gradients (left panel) and percent reduction (right panel) were shown. Mean + standard deviation (SD) is marked. Pre, prior to; post, following balloon aortic valvuloplasty. Reproduced from Reference [84].

The author assessed the immediate results of BAV in sixteen children in the late-1980s [31,42]; the results of a greater number of children (N = 26) were reviewed later [48,63]. In the first 16 children, a reduction of peak systolic pressure gradient across the aortic valve occurred (72 ± 21 vs. 28 ± 13 mmHg; *p* < 0.001) (Figure 17, Figure 18 and Figure 19). In a similar fashion, the left ventricular peak systolic pressure (162 ± 21 vs. 124 ± 18 mmHg; *p* < 0.001) and end-diastolic pressure (13 ± 5 vs. 9 ± 6 mmHg; *p* < 0.01) fell. There was no notable change in cardiac index (3.4 ± 0.5 vs. 3.4 ± 0.4 L/min/m^2^; *p* > 0.1) [42]. In general, the pressure gradients decreased by 60% of pre-valvuloplasty values (Figure 20).

**Figure 21:**
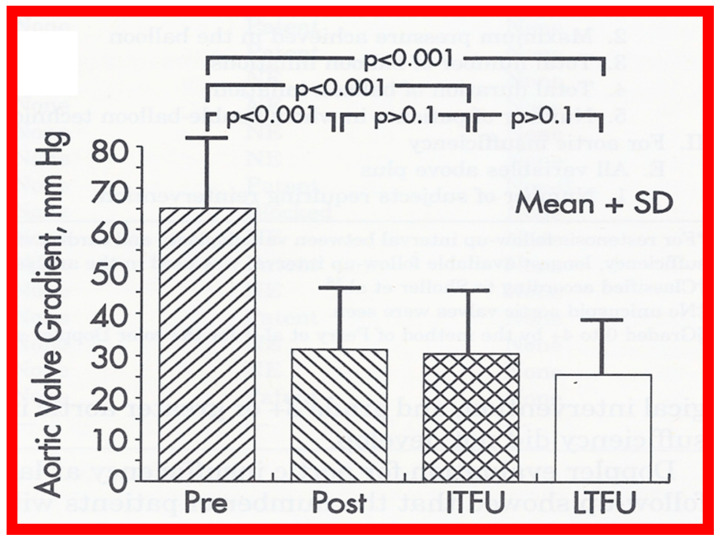
Bar graph showing maximal peak instantaneous Doppler gradients before (Pre) and 1 day after (Post) balloon aortic valvuloplasty and at intermediate-term (ITFU) and late (LTFU) follow-up. There was a significant reduction (*p* < 0.001) in the gradient after balloon aortic valvuloplasty which remained essentially unchanged (*p* > 0.1) at ITFU (12 ± 5 months) and at LTFU (3 to 9 years [mean 6 years]). Doppler-derived maximal peak instantaneous gradients at follow-up continued to be lower (*p* < 0.001) than pre-valvuloplasty gradients. Reproduced from Reference [48].

**Figure 22:**
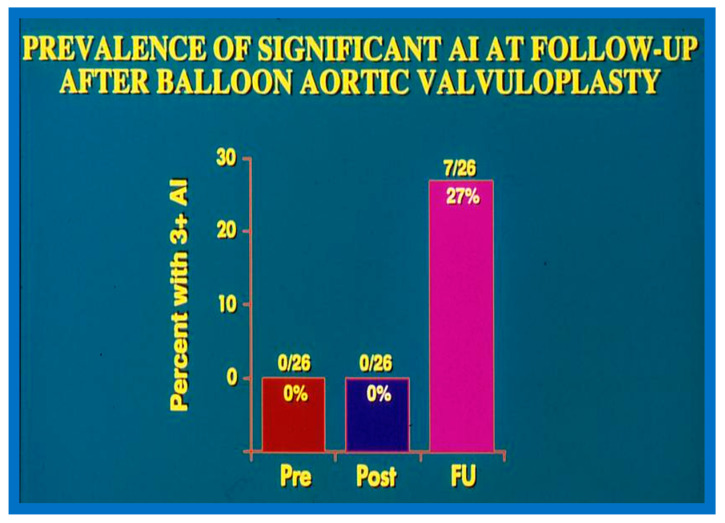
Bar graph demonstrating the prevalence of grade III aortic insufficiency prior to (Pre), immediately following (Post) balloon aortic valvuloplasty, and at late follow-up (FU). No change in aortic insufficiency is seen immediately after balloon valvuloplasty. However, a significant increase occurred at late follow-up. Modified from Reference [83].

In the second cohort consisting of 26 children [48], the immediate outcome was like that of the first cohort as well as those seen by other investigators, as charted elsewhere (Table I of Reference [63]). Peak instantaneous Doppler gradients also decreased like catheter pullback recordings (Figure 21). Significant (3+ or more) aortic insufficiency did not occur (Figure 22).

**Figure 23:**
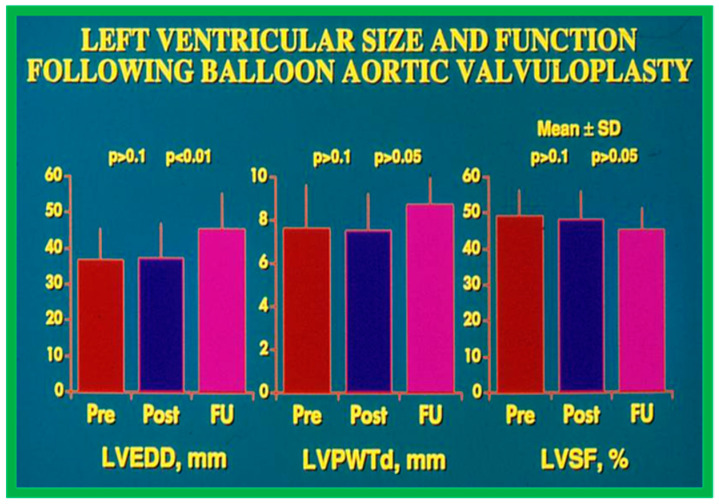
Bar graph demonstrating left ventricular (LV) end-diastolic dimension (EDD) in mm (left panel), LV posterior wall thickness in diastole (PWTd) in mm (middle panel), and LV shortening fraction (SF) in % (right panel) prior to (Pre), on the day after (Post) balloon aortic valvuloplasty, and at late follow-up (FU). Mean + standard deviations (SD) are marked. Note that LVEDD, LVPWTd, and LVSF did not change (*p* > 0.1) immediately after balloon aortic valvuloplasty. At late follow-up the LVEDD increased (*p* < 0.001) while the LVPWTd and LVSF remain unchanged (*p* > 0.05). Reproduced from Reference [116].

Echocardiographic findings disclosed no difference in the 1. LV end-diastolic dimension (36 ± 9 vs. 35 ± 10 mm; *p* > 0.1), 2. The thickness of the LV posterior wall in diastole (7.2 ± 2.1 vs. 7.5 ± 1.9 mm; *p* > 0.1), and 3. LV shortening fraction (50 ± 8 vs. 47 ± 8%; *p* > 0.1) following BAV (Figure 23). However, the Doppler flow velocity magnitudes across the aortic valve (4.0 ± 0.05 vs. 3.0 ± 0.8 m/s; *p* < 0.001) diminished as were the peak instantaneous Doppler gradients (Figure 21). No child from our study subjects required immediate surgical therapy. Immediate results after BAV documented during the decade of 1983–1992 were tabulated (Table I) in our book [63] as well as in other reviews [31,48,63,83] and will not be detailed because of limitations of space.

#### Aortic Stenosis in the Fetus

The description of the results of BAV in fetuses was detailed elsewhere [116] and will not be reviewed here because of the limitation of space.

### 4.3. Aortic Coarctation–Native

**Figure 24:**
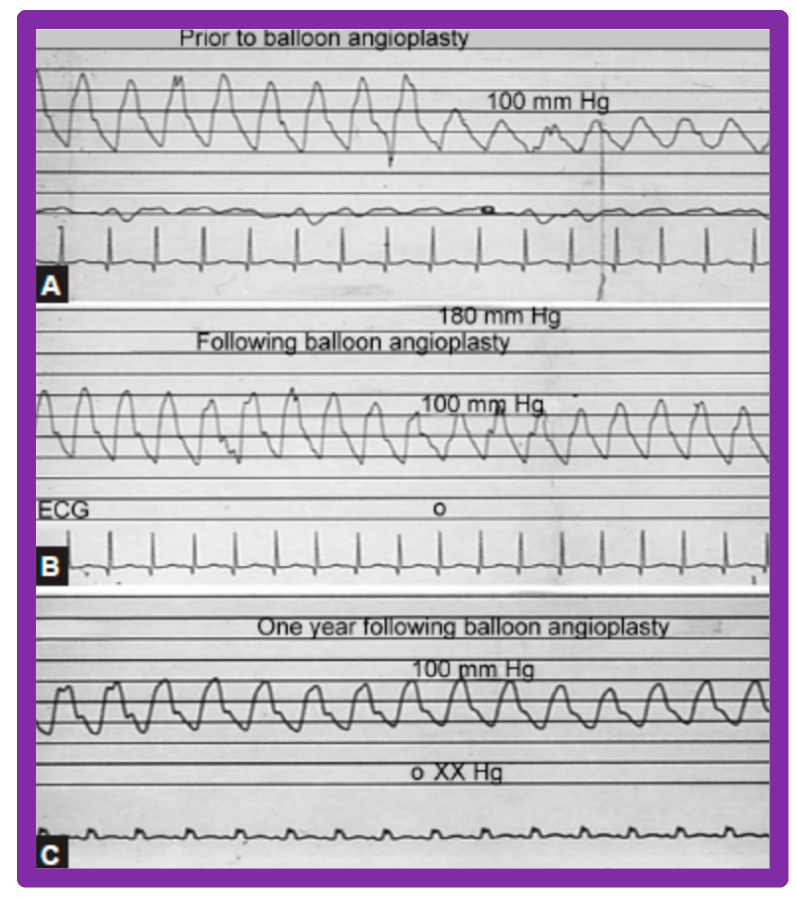
Pressure pullback recordings across the aortic coarctation (**A**) prior to, (**B**) immediately following, and (**C**) 1 year after balloon angioplasty. Note the reduction of the peak-to-peak systolic gradient across the coarctation site (**B**) immediately following balloon angioplasty which (**C**) persisted one year later. Pressures are marked in mmHg. ECG, electrocardiogram. Reproduced from Reference [94].

**Figure 25:**
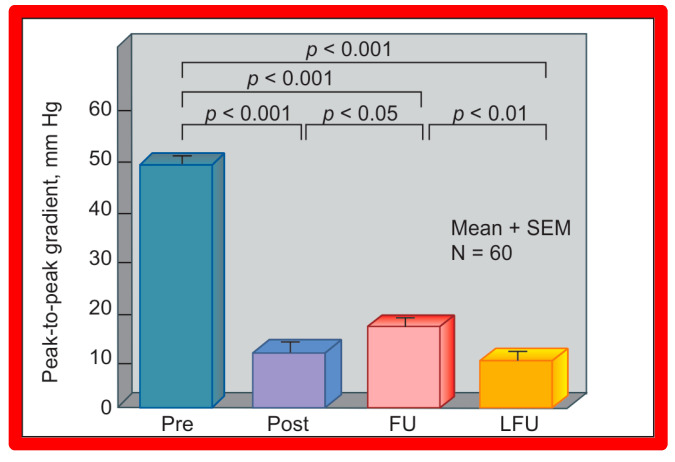
A bar graph of results following balloon angioplasty of native aortic coarctation is shown. Peak-to-peak systolic pressure gradients across the coarctation (shown as mean + SEM (standard error of mean)) decreased significantly (*p* < 0.001) from prior to (Pre) immediately after (Post) balloon angioplasty. However, the gradient increased (*p* < 0.05) slightly at intermediate-term follow-up (FU). However, these gradients continue to be lower (*p* < 0.001) than pre-angioplasty values. At long-term follow-up (LFU) arm-leg peak systolic pressure difference, measured by blood pressures is lower than coarctation gradients prior to (*p* < 0.001) and at intermediate-term follow-up (*p* < 0.01). SEM, standard error of mean; N, number of patients undergoing balloon angioplasty. Reproduced from Reference [94].

**Figure 26:**
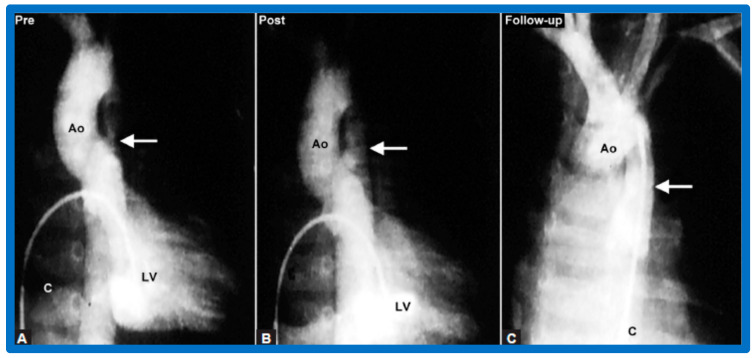
Selected left ventricular (LV) and aortic (Ao) cine-angiographic frames of a nine-month-old baby (**A**) prior to, (**B**) immediately after and (**C**) 1-year following balloon angioplasty are shown. The coarcted aortic segment (arrow) improved remarkably (**B**) after angioplasty (**C**) which continues to be wide open at follow-up. C, catheter. Reproduced from Reference [64].

**Figure 27:**
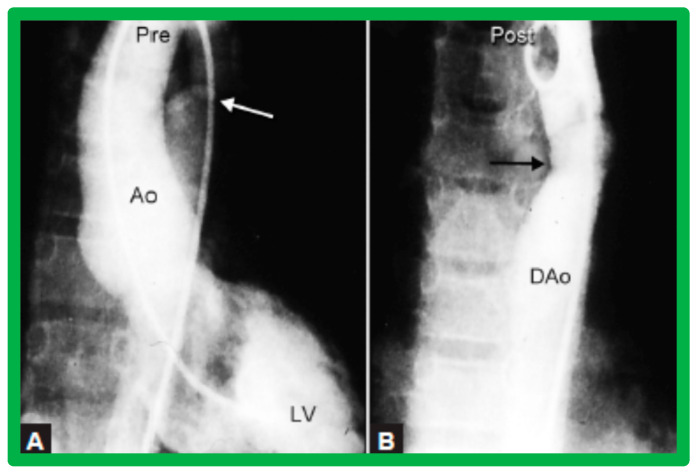
Selected left ventricular (LV) and descending aortic (DAo) cine frames from a posteroanterior view demonstrating (**A**) discrete aortic coarctation (white arrow) in a 10-year-old child (**B**) with remarkable improvement after balloon angioplasty (black arrow). Ao, aorta. Reproduced from Reference [64].

**Figure 28:**
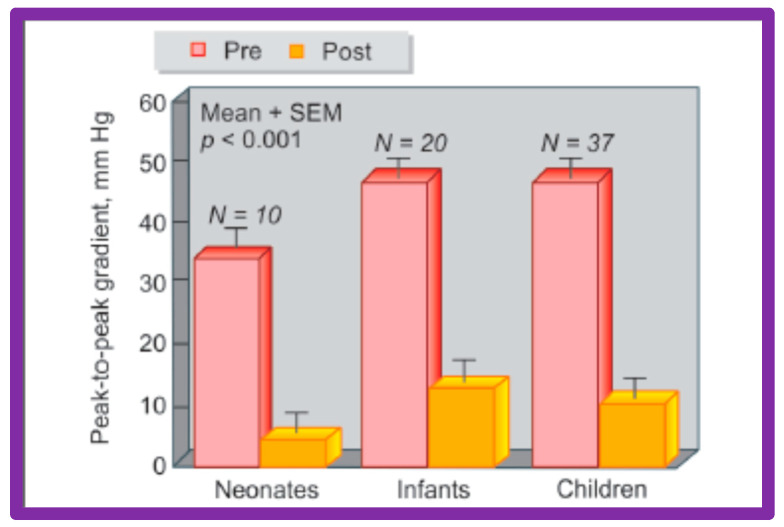
A bar graph of results following balloon angioplasty of native aortic coarctation in different age groups is shown. Peak-to-peak systolic pressure gradients across the coarctation (shown as mean + SEM) decreased significantly (*p* < 0.001) from prior to (Pre-red) immediately after (Post-orange) balloon angioplasty in each age group. SEM, standard error of mean; N, number of patients undergoing balloon angioplasty in each age group. Reproduced from Reference [94].

**Figure 29:**
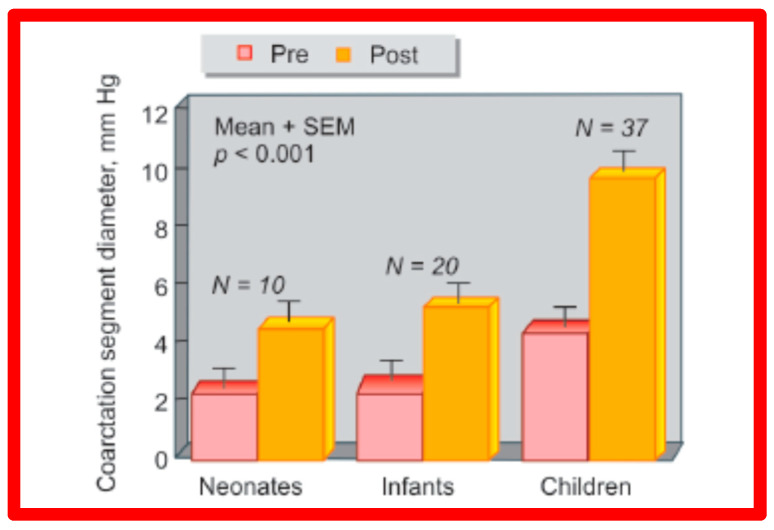
A bar graph of results following balloon angioplasty of native aortic coarctation in different age groups is shown. Diameter of the coarctation segment (shown as mean + SEM) increased significantly (*p* < 0.001) from prior to (Pre-red) immediately after (Post-orange) balloon angioplasty in each age group. SEM, standard error of mean; N, number of patients undergoing balloon angioplasty in each age group. Reproduced from Reference [94].

Immediate results of BA of AC were assessed by the author in the mid-1980s [15,16,43]; subsequently, immediate results of a larger number of children [44,46,47,64] were published. In addition, the results of BA of neonatal and young infant coarctations were subsequently examined [97,98]. A decrease in the pressure gradient across the AC (Figure 24 and Figure 25) and angiographic improvement of coarctation occurred (Figure 9, Figure 10, Figure 26, and Figure 27). Reduction of the pressure gradient (Figure 28) and increase in the diameter of the coarcted aortic segment (Figure 29) were found in all age subsets [46,47,64,137].

**Figure 30:**
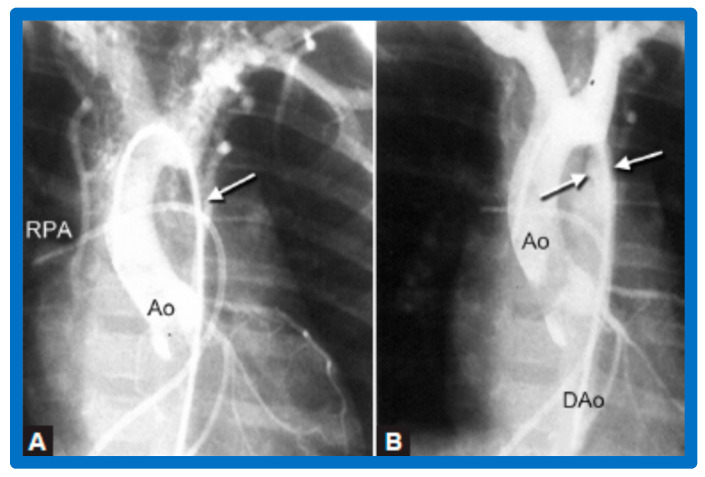
Selected cine-angiographic frames from a posteroanterior view of aortograms (**A**) before and (**B**) after balloon angioplasty, demonstrating aortic coarctation (white arrow) and many collateral vessels in (**A**). (**B**) Immediately after balloon angioplasty, the aortogram shows a marked decrease in collateral vessels. The site of dilated coarctation segment (arrows in (**B**)) is wide open. Additionally, note a better opacification of the descending aorta (DAo) in (**B**) than in (**A**). A catheter is seen in the right pulmonary artery (RPA). Ao, aorta. Reproduced from Reference [64].

We have also documented a reduction of the collateral vessel formation following a BA (Figure 30). The femoral arterial pulses which were either absent or markedly decreased and delayed were felt better after BA. In addition, there was increased pulse volume following BA. Patients who were in CHF got better as did their hypertension. Ventilator-dependent babies could be weaned off the ventilatory support and extubated afterward. Older infants (past the neonatal period) and children were sent home within 24 h after BA. No patient from our study groups needed immediate surgical therapy [46,47,64,137]. Other studies reporting the results of BA of AC were reviewed elsewhere [27,39,47,64,137,138] for the interested reader.

#### Revisit BA in the Neonate and Young Infant

**Figure 31:**
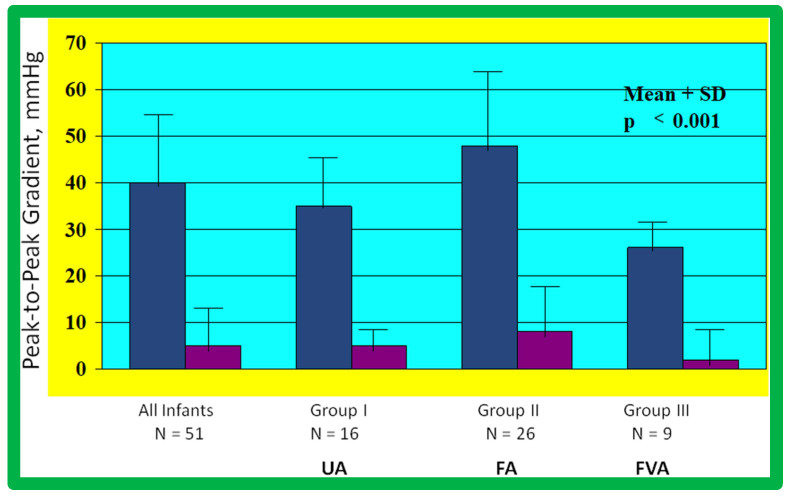
Bar graph showing reduction (*p* < 0.001) of peak-to-peak systolic pressure gradients (in mmHg) across the aortic coarctation after balloon angioplasty. The fall in the gradients was seen for the entire group (left panel) and for all three subgroups, namely trans-umbilical arterial (UA), trans-femoral arterial (FA), and trans-femoral venous anterograde (FVA). Mean + standard deviation (SD) is shown. N represents the number of subjects in each group. Modified from Reference [98].

As reviewed above, the ideal treatment approach of native AC in neonates and young infants is controversial [97,98,102]. We hypothesized that BA of AC in infants less than 3 months of age offers efficacious palliation, defined as avoiding surgical procedures for at least four weeks as well as control of CHF. To test this hypothesis, we scrutinized our practice of BA of AC in babies less than 3 months of age [98]. The data on 51 neonates and infants less than 3 months of age with AC (during a 6.5-year period ending in June 2001) who presented with CHF, hypertension or both were reviewed. BA was undertaken via an umbilical artery (UA), femoral artery (FA), or anterograde femoral venous (FVA) in 16, 26, and 9 babies, respectively. Acute results revealed a decrease in peak gradient across the AC from 40 ± 17 mmHg to 5.4 ± 6.1 mmHg (*p* < 0.001) (Figure 31, left panel) and improved coarcted segment diameter from 2.2 ± 0.5 mm to 5.6 ± 0.8 mm (*p* < 0.001). There was also a noticeable improvement in the symptoms. The route of access used to perform BA did not have an influence on the effectiveness of the procedure (Figure 31, right panels).

**Figure 32:**
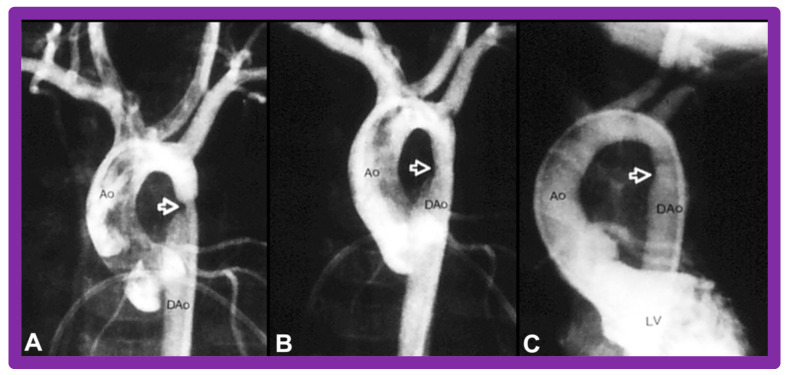
Selected aortic (Ao) and left ventricular (LV) cine-angiographic frames of a one-month-old baby prior to (**A**), immediately after (**B**), and 1 year following (**C**) balloon angioplasty are shown. The coarcted aortic segment (arrowhead) shown in “**A**” improved remarkably after angioplasty (arrowhead in (**B**)) which continues to be wide open at follow-up (arrowhead in (**C**)). DAo, descending aorta. Reproduced from Reference [131].

During follow-up, surgery to alleviate residual aortic narrowing was required in 4 babies at 5, 21, 24, and 28 days following BA, respectively. Hence, successful palliation was accomplished in the outstanding 47 babies (92%). During a median follow-up of three years (5 months to 5.5 years), most babies were well and had decent angiographic improvement (Figure 32). However, twenty-two babies (~50%) were judged to have re-coarctation, defined as a peak-to-peak gradient greater than 20 mmHg. Fourteen babies had repeat BA and eight babies had surgery two to ten months (median of three months) following the original BA. The reason for repeat procedures was hypertension in all infants. Repeat BA in these 14 babies resulted in a decrease in peak systolic pressure gradients (54 ± 20 mmHg vs. 9 ± 7 mmHg; *p* < 0.001). At the last follow-up (median 3 years, range, 0.5 to 5.5 years), arm blood pressures were low at 98 ± 11 mmHg and an arm-to-leg blood pressure difference was 4 ± 6 mmHg.

**Figure 33:**
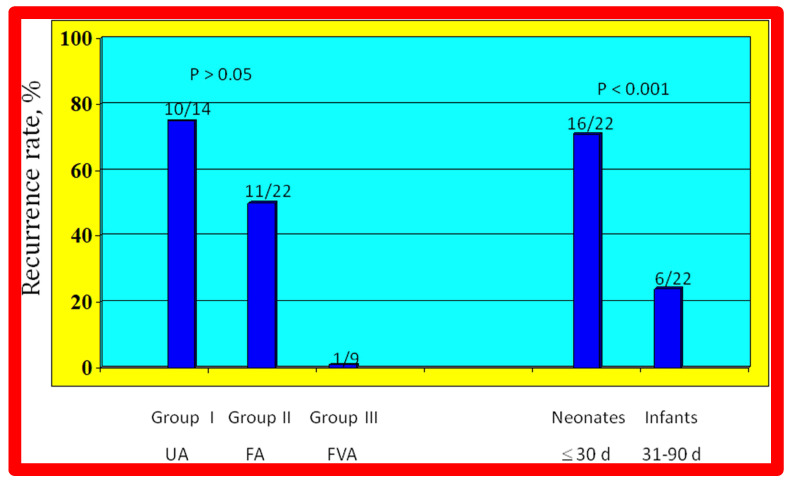
Bar graph demonstrating re-coarctation rates during follow-up after balloon angioplasty of native coarctation in infants. The rate of recurrence is not related (*p* > 0.05) to the route through which balloon angioplasty was performed (left panel). However, when the patients were divided into neonates (≤30 days) and infants between 31 and 90 days, the rate of recurrence was significantly higher (*p* < 0.001) in neonates than in infants (right panel). Number of subjects with recurrence/number of subjects in that group is shown on the top of each bar. The data indicate that age at angioplasty plays a major role in re-coarctation and not the route of balloon angioplasty. d, days; FA, femoral artery; FVA, femoral venous, anterograde; UA, umbilical artery. Modified from Reference [98].

The re-coarctation rates in the UA, FA, and FVA subsets were similar (*p* > 0.05) (Figure 33, left panel). However, babies ≤ 30 days old had higher (sixteen of 22 {72%} vs. six of 22 {28%}; *p* < 0.001) prevalence of re-coarctation than babies 31 to 90 days old (Figure 33, right panel). Thus, the re-coarctation prevalence appears to be correlated to the age at BA rather than to the route by which the BA was accomplished.

Based on these data, it was concluded that effective palliation is attained with BA in all 3 groups, and our hypothesis was confirmed. We believe that BA is an excellent substitute for surgery in the treatment of native AC in neonates and young infants [97,98]. However, some cardiologists advocate not using BA in neonates but may accept it for babies older than 3 months.

### 4.4. Aortic Coarctation, Postsurgical

**Figure 34:**
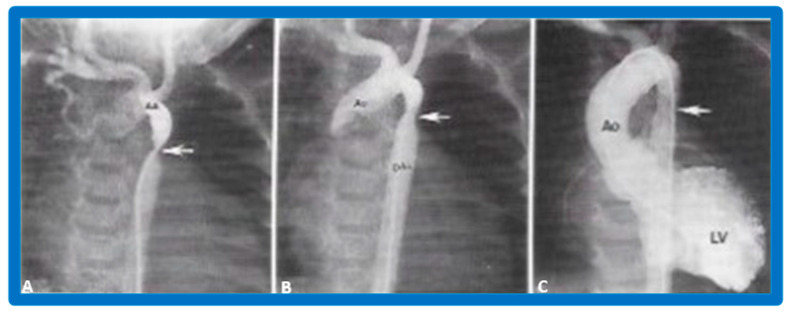
Aortic arch (AA) cine-angiogram in posteroanterior projection prior to (**A**), immediately after (**B**) balloon angioplasty demonstrating severe post-operative aortic re-coarctation (arrow in (**A**)). Balloon angioplasty resulted in an improvement in angiographic diameter (arrow in (**B**)). Repeat left ventricular (LV) cine twelve months later continues to show wide open aortic segment (arrow in (**C**)). DAo, descending aorta; LV, left ventricle. Reproduced from Reference [65].

**Figure 35:**
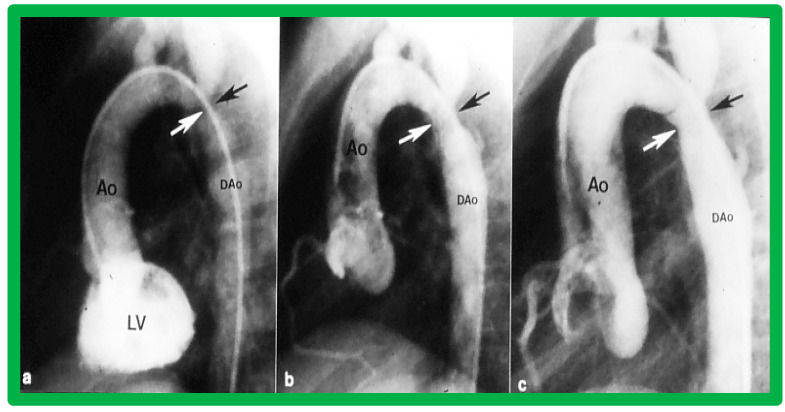
Left ventricular cine-angiogram in lateral projection demonstrating severe post-operative aortic coarctation (arrows in (**a**)). Balloon angioplasty resulted in an improvement in angiographic diameter (arrows in (**b**)). Repeat aortic (Ao) root cine one year later continues to show wide open aortic segment (arrows in (**c**)). DAo, descending aorta. Reproduced from Reference [65].

**Figure 36:**
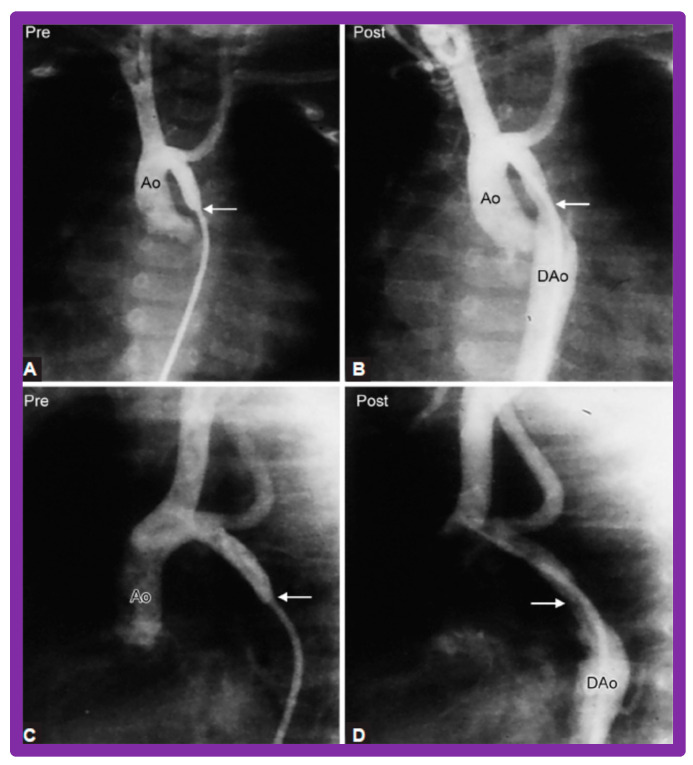
Selected aortic (Ao) cine-angiographic frames in posteroanterior and lateral views of a young child (**A**,**C**) prior to and (**B**,**D**) immediately after balloon angioplasty of postsurgical aortic re-coarctation. The coarcted aortic segment (arrows) (**B**,**D**) improved remarkably after angioplasty. DAo, descending aorta. Reproduced from Reference [65].

Immediate results of BA of postsurgical AC were evaluated by the author in a limited number of patients initially [27,45,46,65]; subsequently, immediate results of a larger number of children [49] were available. In the initial 11 patients, BA was performed during a 70-month period ending July 1991; they developed re-coarctation six months to 7.5 years following surgery for native AC. BA resulted in the reduction of the coarctation gradient from 48 ± 25 to 14 ± 8 mmHg (*p* < 0.001) and an increase in coarcted aortic segment diameter from 3.1 ± 1.3 to 6.2 ± 1.2 mm (*p* < 0.01). Clinical improvement was demonstrated in all patients [65]. Angiographic examples are shown in Figure 34, Figure 35 and Figure 36.

**Figure 37:**
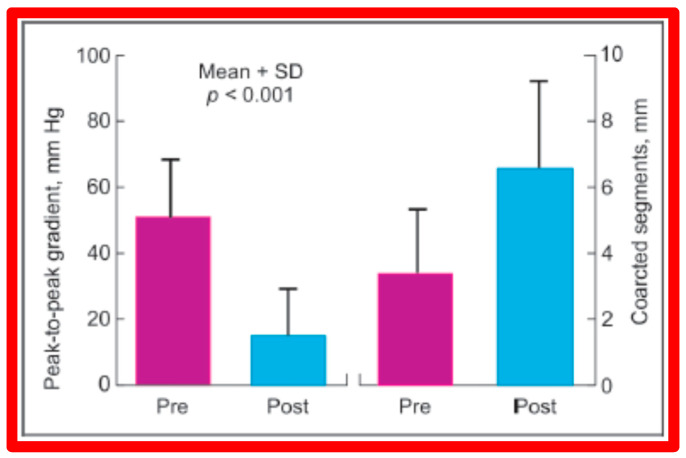
A bar graph of results following balloon angioplasty of postsurgical aortic re-coarctation is shown. Peak-to-peak systolic pressure gradients across the coarctation decreased significantly (*p* < 0.001) from prior to (Pre) to immediately after (Post) balloon angioplasty. Similarly, the diameter of the coarcted aortic segment increased significantly (*p* < 0.001) after balloon angioplasty. Mean + SD (standard deviation) is shown. Modified from Reference [49].

**Figure 38:**
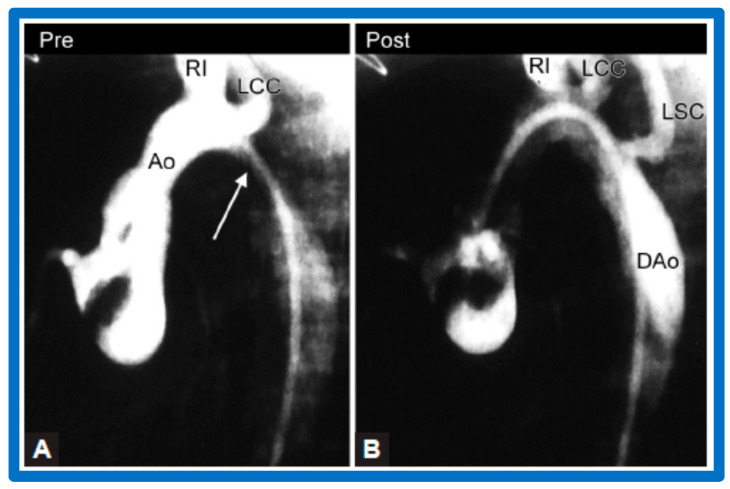
Selected aortic (Ao) cine-angiographic frames in lateral view of a young child (**A**) prior to and (**B**) immediately after balloon angioplasty of postsurgical aortic re-coarctation. The coarcted aortic segment (white arrow in (**A**)) (**B**) improved remarkably after angioplasty. DAo, descending aorta; LCC, left common carotid artery; LSC, left subclavian artery; RI, right innominate artery. Reproduced from Reference [94].

**Figure 39:**
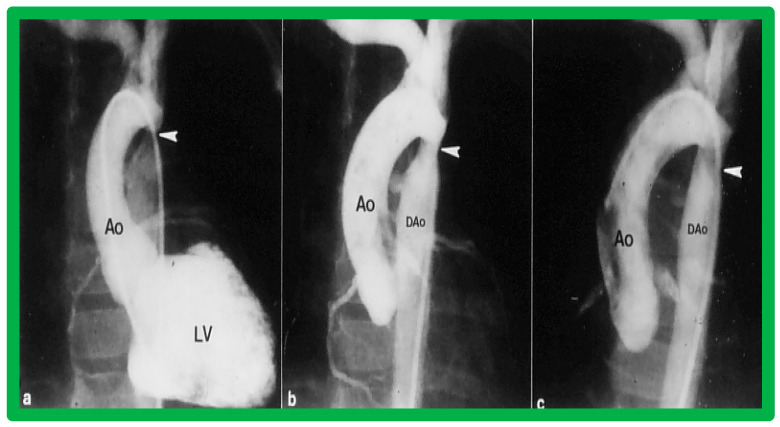
Left ventricular cine-angiogram in anteroposterior projection demonstrating severe post-operative aortic coarctation (arrowhead in (**a**)). Balloon angioplasty resulted in improvement in angiographic diameter (arrowhead in (**b**)). Repeat aortic (Ao) root cine one year later continues to show wide open aortic segment (arrowhead in (**c**)). DAo, descending aorta. Reproduced from Reference [49].

In a subsequent study involving 33 patients [49], an equally impressive reduction in the peak-to-peak systolic pressure gradient across the re-coarctation site (48 ± 22 vs. 13 ± 15 mmHg; *p* < 0.01) and an increase in the coarcted aortic segment size (3.2 ± 1.4 vs. 6.4 ± 2.3 mm; *p* < 0.001) occurred (Figure 37). Additional angiographic examples are shown in Figure 38 and Figure 39.

**Figure 40:**
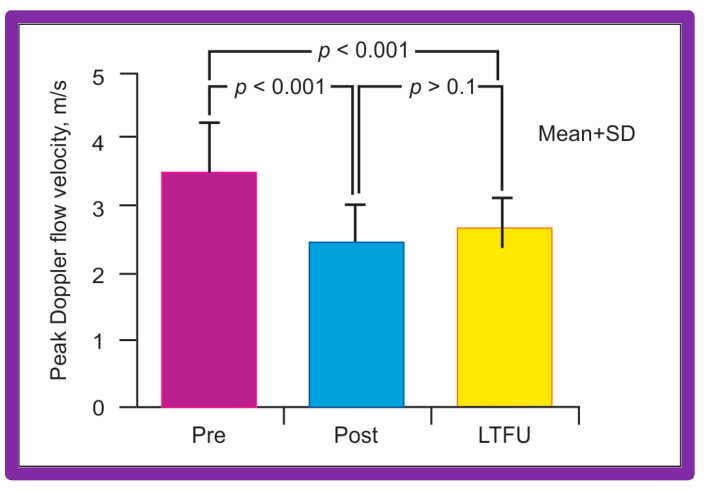
Bar graph shows Doppler flow velocities across the coarctation site prior to (Pre) and immediately after (Post) balloon angioplasty of postsurgical aortic re-coarctation and at long-term follow-up (LFTU). Note significant (*p* < 0.001) fall in Doppler flow velocities after balloon angioplasty. At LFTU, the Doppler flow velocities continue to be lower (*p* < 0.001) than those prior to balloon angioplasty. Mean + SD (standard deviation) is shown. Modified from Reference [94].

Doppler flow velocities across the coarctation site decreased from 3.5 ± 0.8 to 2.4 ± 0.6 m/s (*p* < 0.001) as did the Doppler peak instantaneous gradients (47 ± 22 vs. 20 ± 10 mmHg; *p* < 0.001) after BA in a manner like those of catheterization gradients (Figure 40). Diastolic extension of the Doppler signal that was seen prior to BA was no longer persistent in the post-BA studies.

Most of the children were sent home within 48 h after BA. Two (6%) out of 33 children needed surgery to address complications [49]. The observed improvement described above is independent of the type of initial surgery (end-to-end anastomosis, subclavian flap angioplasty, patch angioplasty, or repair of interrupted aortic arch) [45,49,65]. Our immediate results are basically like those described by other cardiologists which were tabulated in our previous publications [45,49,65] as well as those of the VACA Registry [111]. Likewise, satisfactory outcomes of BA have been seen in aortic re-coarctations that happen after a Norwood operation [49,139,140] and after cardiac transplantation [141].

## 5. Short-Term Results

Short-term, defined as six to 24 months, following balloon dilatation will be examined for each of the lesions under review.

### 5.1. Pulmonary Stenosis

**Figure 41:**
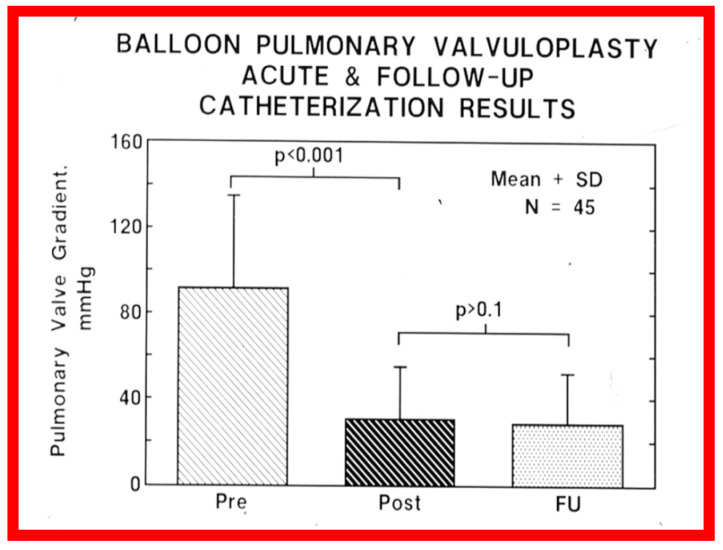
Bar graph showing the immediate and follow-up peak-to-peak pulmonary valve systolic pressure gradients in 45 unselected patients who underwent cardiac catheterization at a mean of 11 months following balloon pulmonary valvuloplasty. Note the significant (*p* < 0.001) fall in the gradient immediately after (Pre vs. Post) balloon dilatation, which remained unchanged (*p* > 0.1) at follow-up (FU). N, number of patients. The mean + standard deviation (SD) is shown. Reproduced from Reference [62].

**Figure 42:**
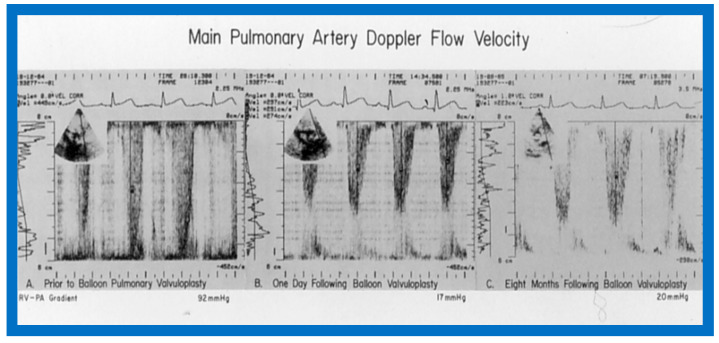
Doppler flow velocity recordings from the main pulmonary artery prior to (**A**), one day (**B**) and eight months (**C**) after balloon pulmonary valvuloplasty are shown. Note that there was a remarkable decrease in the peak flow velocity and calculated gradient (92 mmHg vs. 17 mmHg) one day after the procedure, and these remained low (20 mmHg) at follow-up. Reproduced from Reference [69].

**Figure 43:**
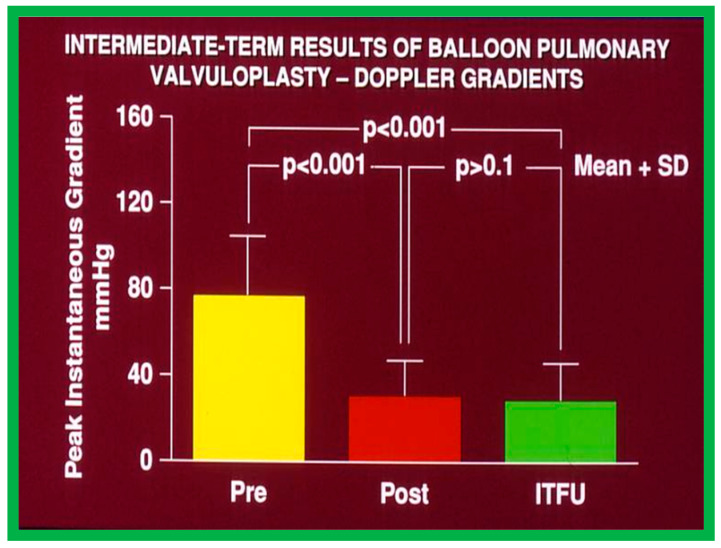
Bar graph demonstrating maximum peak instantaneous Doppler gradients prior to (Pre) and one day following (Post) balloon pulmonary valvuloplasty, and at intermediate-term follow-up (ITFU) (Short-term). Note the significant reduction (*p* < 0.001) after valvuloplasty which remains unchanged (*p* > 0.1) at ITFU. The mean + standard deviation (SD) is shown. Modified from Reference [50].

**Figure 44:**
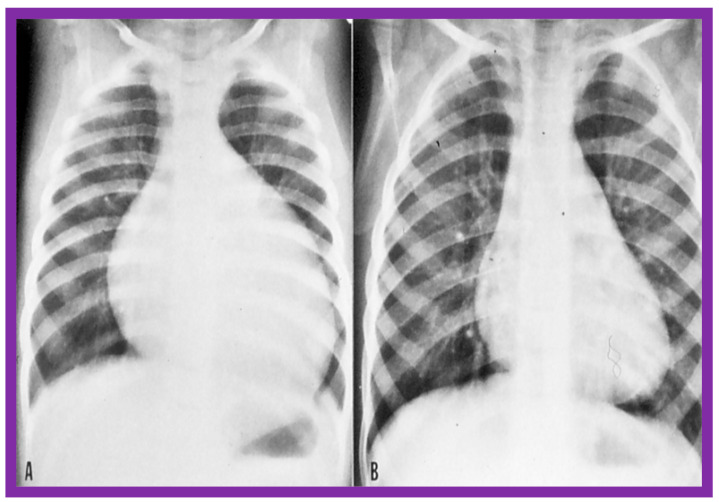
The size of the heart prior to (**A**) and 1 year following (**B**) balloon pulmonary valvuloplasty, demonstrating the decreased size of the heart at follow-up. Reproduced from Reference [136].

**Figure 45:**
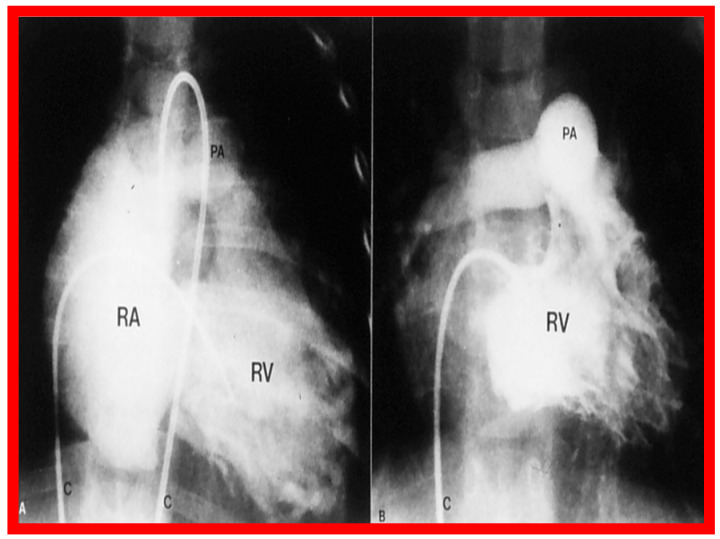
Right ventricular (RV) cine-angiographic frames prior to (**A**) and 1 year following (**B**) balloon pulmonary valvuloplasty, demonstrating the almost complete disappearance of tricuspid insufficiency. C, catheter; PA, pulmonary artery, RA, right atrium. Reproduced from Reference [62].

**Figure 46:**
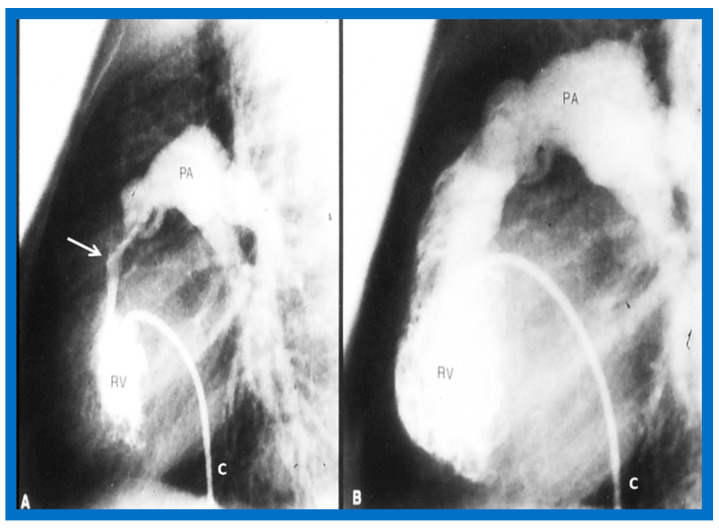
Selected cine frames from right ventricular (RV) angiogram in lateral view, showing severe infundibular stenosis (arrow) (**A**) immediately after balloon pulmonary valvuloplasty. Note the wide open right ventricular outflow tract (**B**) at cardiac catheterization 10 months after balloon valvuloplasty. The peak-to-peak pulmonary valvar pressure gradient at follow-up catheterization was 20 mmHg; there was no infundibular gradient. C, catheter; PA, pulmonary artery. Reproduced from Reference [69].

**Figure 47:**
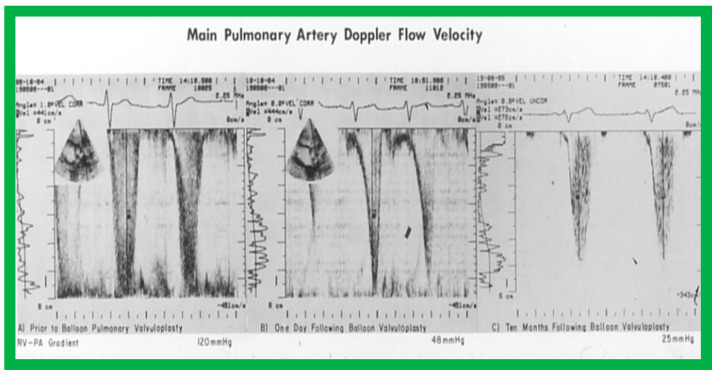
The main pulmonary artery Doppler flow velocities prior to (**A**), one day (**B**), and ten months (**C**) following balloon pulmonary valvuloplasty are shown. Note that there is a significant fall in the peak flow velocity immediately after valvuloplasty, but a moderate (48 mmHg) gradient that has a characteristic triangular pattern, highly suggestive of infundibular obstruction (corresponding to Figure 20 left) persisted. At the 10-month follow-up, the flow velocity has markedly diminished, indicating the resolution of the infundibular obstruction (corresponding to Figure 20 right). The residual calculated gradients are shown at the bottom of each panel. Reproduced from Reference [69].

At short-term follow-up, the peak pressure gradients across the pulmonary valve stayed improved when compared with pre-BPV gradients and did not significantly change when compared to immediate post-BPV gradients for the entire cohort. This decrease in the gradients was shown by both catheterization-quantified peak-to-peak gradients (Figure 41) and by Doppler-calculated peak instantaneous gradients (Figure 42 and Figure 43) [25,41,50,62,66,136]. The heart size on a chest X-ray (Figure 44), right-to-left shunt across the atrial septum (Figure 16), right ventricular function, the degree of tricuspid insufficiency (Figure 45), and the RV infundibular obstruction (Figure 46 and Figure 47) improved at short-term follow-up [25,41,50,62,66,136]. There was only a minimal increase in the degree of pulmonary insufficiency at short-term follow-up [50].

**Figure 48:**
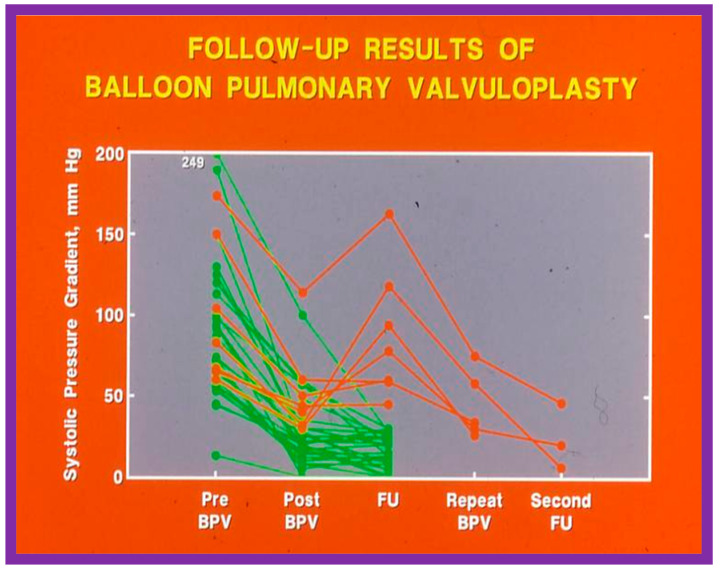
Line graph showing pulmonary valve gradients prior to (Pre) and immediately following (Post) balloon pulmonary valvuloplasty (BPV), and at follow-up (FU). The patients with good results are shown in green while those with poor results are shown in orange. Repeat BPV was performed in five patients; the gradients fell and remained low at the second FU. Modified from Reference [25].

In spite of the seemingly good results for the entire cohort, the development of restenosis of the pulmonary valve (defined as a gradient of more than 50 mmHg) was observed in about 10% of children (Figure 48) at short-term follow-up [50,142]. Discussion of causes of restenosis and the feasibility repeat BPV in addressing the restenosis will be reviewed in the ensuing sections.

#### 5.1.1. Causes of Restenosis

**Figure 49:**
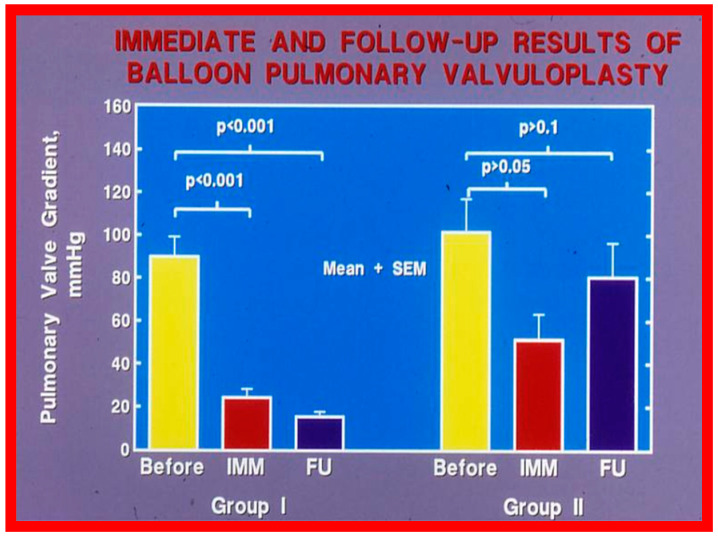
Bar graph showing the immediate (IMM) and follow-up (FU) results of balloon pulmonary valvuloplasty in Group I (with good results) (left panel) and in Group II (with poor results) (right panel). In Group I, the pulmonary valve gradient decreased significantly (*p* < 0.001) immediately after valvuloplasty and remained low (*p* < 0.001) at follow-up. In Group II, the pulmonary valve gradient fell slightly (*p* > 0.05) immediately after valvuloplasty and returned to the pre-valvuloplasty values (*p* > 0.1) at follow-up. The mean + standard error of mean (SEM) is shown. Reproduced from Reference [75].

Recurrence of stenosis after BPV was observed at follow-up as discussed in the preceding section (Figure 48). The causes of restenosis following BPV were examined by analyzing the follow-up outcomes of 36 patients [142]. The study cohort was split into two groups: Group I—29 patients with good results (residual peak systolic pressure gradients < 30 mmHg), and Group II—7 patients with bad results (residual peak systolic pressure gradients ≥ 30 mmHg). In Group I patients, the peak pulmonary valve gradients were reduced (90 ± 48 vs. 25 ± 19 mmHg; *p* < 0.001) at the time of BPV; these gradients further decreased to 16 ± 10 mmHg (*p* < 0.01) at short-term follow-up evaluation (Figure 49, left panel). No child in this group needed re-intervention. In Group II patients, the peak gradients across the pulmonary valve were slightly reduced (102 ± 44 vs. 52 ± 30 mmHg; *p* > 0.05) at the time of BPV; these gradients increased to 81 ± 41 mmHg at short follow-up evaluation (Figure 49, right panel). Five of these patients underwent repeat balloon BPV resulting in a significant (*p* < 0.01) drop in the pulmonary valve peak pressure gradients. The other two patients with residual gradients of 45 and 60 mmHg, respectively, were periodically followed without re-intervention, as per the wishes of their physician. The pressure gradient information of these patients is shown in Figure 48.

**Figure 50:**
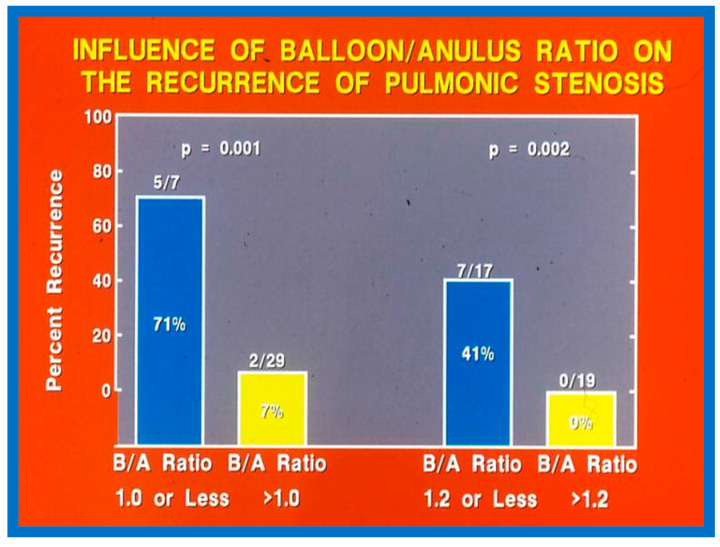
Bar graph demonstrating the influence of the balloon/annulus ratio (B/A) on rates of recurrence of pulmonary valve stenosis after balloon pulmonary valvuloplasty. Note that the rate of restenosis decreases as the balloon/annulus ratio increases (*p* = 0.001 to 0.002). Percentages are marked within the bars and actual numbers are shown on the top of each bar. Modified from Reference [62].

**Figure 51:**
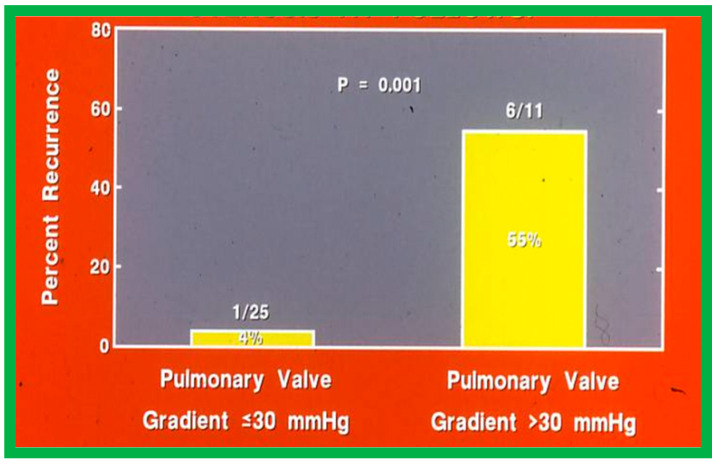
Bar graph like Figure 50, except that the immediate post-valvuloplasty peak-to-peak pulmonary valve gradients are used instead of balloon/annulus ratios. Note the higher (*p* = 0.001) rate of recurrence at follow-up when the gradient is >30 mmHg. Percentages are marked within the bars and actual numbers are shown on the top of each bar. Modified from Reference [62].

Fourteen different biographic, anatomic, physiologic, and technical variables were analyzed by multivariate logistic regression testing to pinpoint the factors producing the reappearance of stenosis [142]. The age at BPV, length of follow-up, the incidence of pulmonary valve dysplasia, existence of valve annulus hypoplasia, presence of infundibular PS, the prevalence of RV hypoplasia, magnitude of RV peak systolic pressure, gradients across the pulmonary valvar and RV infundibulum, number of balloon inflations, the pressure of balloon inflation, and duration of balloon inflation were similar (*p* > 0.1) among the two groups. These data were displayed in Tables I, II, and III of our publication [143] for the interested reader. Stepwise logistic regression evaluation detected two factors responsible for the development of restenosis: 1. A balloon/annulus (B/A) ratio less than 1.2 and 2. An immediate post-BPV pulmonary valve peak pressure gradient of more than 30 mmHg. The correlation between the B/A ratio (Figure 50) and immediate post-BPV peak pressure gradients (Figure 51) on the one hand and the reappearance of PS at follow-up on the other is displayed in Figure 50 and Figure 51. The frequency of restenosis diminishes (*p* = 0.001 to 0.002) as the B/A ratio increases. When the B/A ratio is greater than 1.2, there were no recurrences (Figure 50). Immediate post-BPV peak pulmonary valve gradients greater than 30 mmHg are linked with a greater frequency of recurrence (*p* = 0.001) when compared with post-BPV gradients less than 30 mmHg (Figure 51).

It was concluded a B/A ratio ≤ 1.2 is likely to be the cause for the reappearance of PS at short-term follow-up and such restenosis may be predicted by an immediate post-BPV pressure gradient across the pulmonary higher than 30 mmHg [142]. Consequently, we advocate using balloons large enough to reduce the valvar (not infundibular) pressure gradients to less than 30 mmHg during BPV. In a later study [50] assessing the long-term outcomes in 80 patients, the risk factors for restenosis were precisely identical to the findings of our first investigation [142]. Furthermore, in a multi-institutional VACA Registry study [143], the B/A ratio and immediate post-BPV pulmonary valve gradient were also seen to be predictive of restenosis.

Now that the influence of balloon diameter in restenosis is well recognized, most interventionalists utilize balloons bigger than the pulmonary valve annulus for performing BPV. Consequently, the causes for restenosis at the current time are more likely to be pathologic anomalies, namely, hypoplasia pulmonary valve annulus, pulmonary valve leaflet dysplasia, and supravalvar pulmonary artery stenosis [75].

#### 5.1.2. Feasibility of Repeat BPV to Address Recurrence of PS

**Figure 52:**
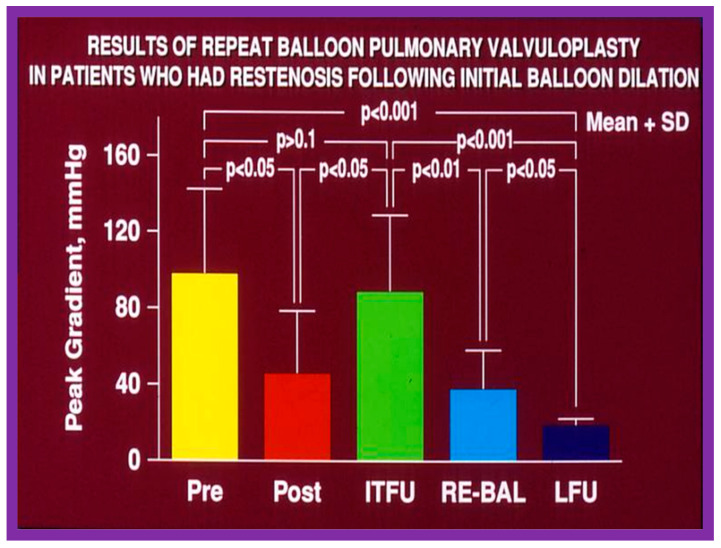
Results of repeat balloon pulmonary valvuloplasty of patients who had restenosis after initial balloon valvuloplasty. The initial gradients were reduced (*p* < 0.05) significantly after valvuloplasty (pre vs. post) but returned toward pre-valvuloplasty values (*p* > 0.1) at intermediate-term follow-up (ITFU). Repeat valvuloplasty (RE-BAL) again reduced the gradient (*p* < 0.01), which had decreased further (*p* < 0.05) at long-term follow-up (LFU) and continued to be lower than the gradients prior to the first (*p* < 0.001) and second (*p* < 0.001) balloon procedures. The mean + standard deviation (SD) is shown. Modified from Reference [144].

As reviewed in the preceding sections, the reappearance of PS following BPV has been documented. The feasibility and effectiveness of repeat BPV in relieving recurrent stenosis were studied by the author [144]. During a 10-year period between 1983 and 1993, 85 patients with PS had BPV; there was a decrease in peak gradients across the pulmonary valve (91 ± 41 mmHg vs. 25 ± 19 mmHg; *p* < 0.01). Residual pulmonary valve gradients at the mid-term follow-up (10.4 ± 7.3 months) of 80 patients were 26 ± 26 mmHg; these gradients are lower (*p* < 0.01) for the entire cohort. Nevertheless, when the data on each patient were examined, nine of the 80 patients (11%) were found to have a recurrence of pulmonary valve stenosis, defined as residual peak-to-peak gradients of more than 50 mmHg. This subset of children had repeat BPV to address recurrent obstruction. In this subset of children, the peak systolic pressure gradients across the pulmonary valve were reduced from 98 ± 45 mmHg to 46 ± 33 mmHg (*p* < 0.05) at the time of the first BPV (Figure 52, left panel). Re-evaluation at a mean of 11 months after the BPV procedure showed residual gradients of 89 ± 40 mmHg, an increase (*p* < 0.05) when contrasted with immediate post-BPV gradients (Figure 52, middle panel). BPV was repeated resulting in a decrease (*p* < 0.01) of peak gradients across the pulmonary valve (89 ± 40 mmHg vs. 38 ± 20 mmHg; *p* < 0.01) (Figure 52). Echo-Doppler examination 2 to 6.5 years after the second BPV indicated good results; the residual peak instantaneous Doppler gradients were 24 ± 13 mmHg (Figure 52).

Based on these data we concluded that repeat BPV is feasible and effective in providing relief of re-obstruction that ensued after initial BPV [144].

### 5.2. Aortic Stenosis

**Figure 53:**
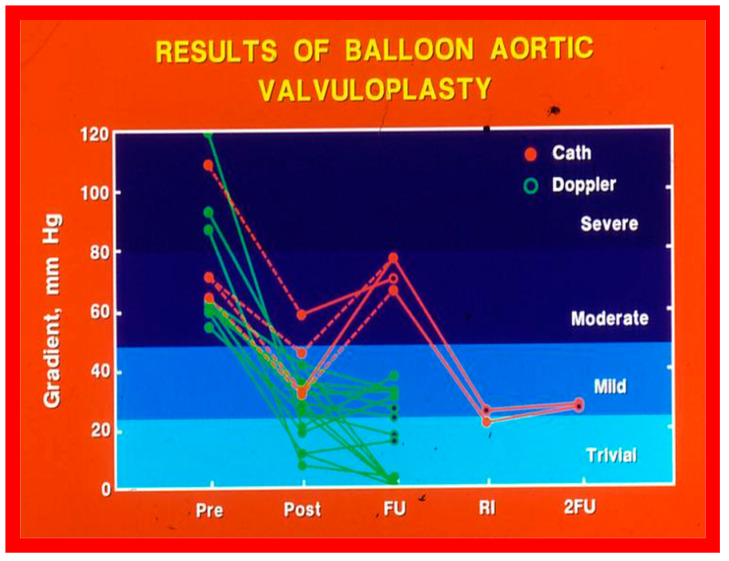
Line graph showing aortic valve peak-to-peak systolic pressure gradients prior to (Pre), immediately following (Post) and at follow-up (FU) after balloon aortic valvuloplasty. Patients with good results are shown in green while those with poor results are shown in orange. Re-intervention (RI) (balloon valvuloplasty) was performed in some patients and the gradients fell. On further follow-up (2FU), the residual gradients remained low. When the severity of the gradients was examined, the severity grade of the stenosis decreased in all patients going from severe to moderate, mild or trivial, and from moderate to mild or trivial. Reproduced from Reference [116]. Solid green lines indicate good results; Orange lines indicate poor results.

At short-term follow-up, peak aortic valve gradients either did not change or increased slightly when compared to immediate results; this was demonstrated by both cardiac catheterization-measured (41 ± 23 mmHg) (Figure 19) and by Doppler-derived peak instantaneous gradients (31 ± 15 mmHg) (Figure 21). However, these gradients remain lesser than pre-BAV gradients [48]. However, if the residual aortic valve gradient of each patient is separately evaluated, recurrence of obstruction, defined as a peak gradient greater than 50 mmHg was observed in 6 (23%) children (Figure 53). Four of these patients had surgical aortic valvotomy during our early experience and two children’s restenosis was addressed by repeat BAV at a median follow-up of 9 months. The degree of AI did not change at short-term follow-up [48]. Short-term follow-up outcomes reported by other investigators were like those of ours; these data were reviewed elsewhere [31,48,63,83] and were presented in Table II of our book chapter [63] for the interested reader.

#### 5.2.1. Causes of Restenosis

**Figure 54:**
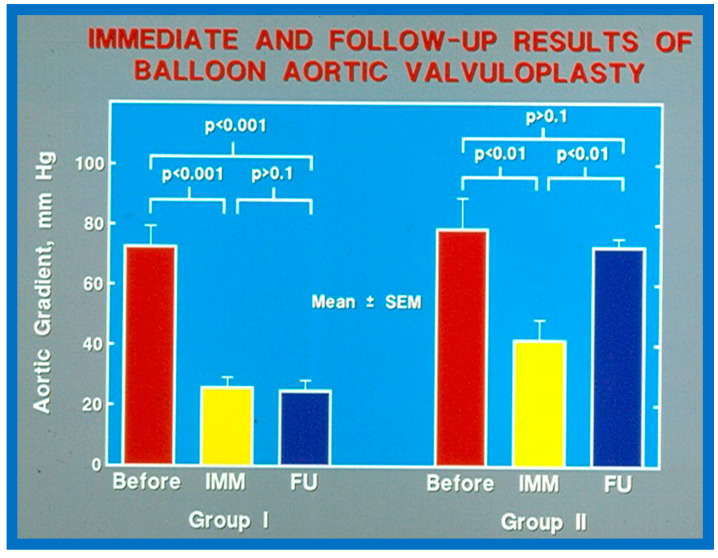
Bar graph showing immediate (IMM) and follow-up (FU) results of balloon aortic valvuloplasty in Group I with good results (left panel) and in Group II with poor results (right panel). In Group I with good results, the aortic valve gradient decreased significantly (*p* < 0.001) immediately after valvuloplasty and remained low (*p* < 0.001) at follow-up. In Group II with poor results, the aortic valve gradient fell (*p* < 0.01) immediately after valvuloplasty and returned to pre-valvuloplasty values (*p* > 0.1) at follow-up. Mean + standard error of mean (SEM) is shown. Reproduced from Reference [116].

As reviewed in the preceding section, recurrence of stenosis happens following BAV (Figure 53). The causes for restenosis following BAV were investigated by studying the follow-up results of 16 patients [42]. First, these 16 patients were divided into two groups: Group I with good results, defined as peak aortic valve pressure gradients < 50 mmHg at follow-up (N = 12), and Group II with poor results, defined as peak gradients > 50 mmHg (N = 4). In Group I children, the peak aortic valve pressure gradient was lowered (70 ± 21 vs. 24 ± 11 mmHg; *p* < 0.001) at the time of BAV; these values remained unchanged (26 ± 10 mmHg; *p* > 0.1) at short-term follow-up (Figure 54; left panel). None of the children in this cohort required further treatment. In Group II patients, the peak gradient across the aortic valve was lowered from 79 ± 20 mmHg to 42 ± 13 mmHg (*p* < 0.001) after BAV. Nonetheless, at short-term re-assessment, the residual peak aortic valve gradient increased significantly (73 ± 5 mmHg; *p* < 0.001) (Figure 54; right panel). All four patients in this cohort had effective relief of the aortic valve obstruction either by surgical valvotomy (N = 2) or by second BAV (N = 2) [42].

**Figure 55:**
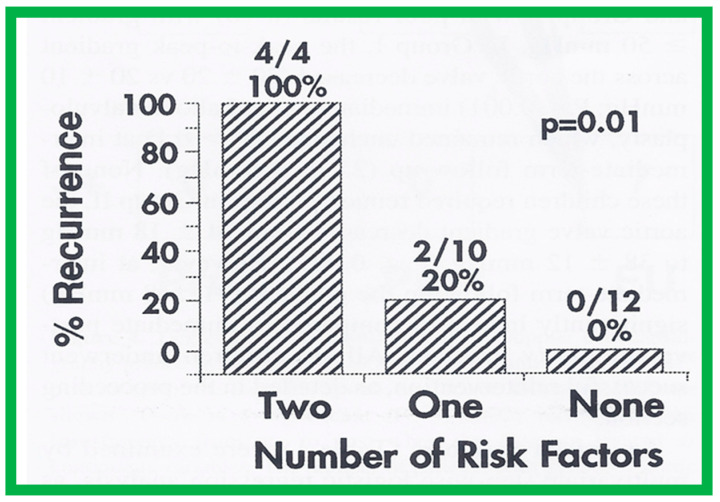
Bar graph demonstrating the influence of multiple risk factors on rates of recurrence of aortic stenosis after balloon aortic valvuloplasty. Note that the larger the number of risk factors, the greater is the probability for restenosis. Percentages and actual numbers are shown on the top of each bar. Reproduced from Reference [83].

Seventeen separate parameters, as listed in Tables I, II, and III of [42] were examined by multivariate stepwise logistic regression analysis, as described in previous publications [42,141] to identify factors that can predict the reappearance of stenosis in Group II patients. This appraisal identified the patient’s age < 3 years at the time of BAV and immediate post-BAV peak aortic valve peak gradient ≥ 30 mmHg as predictors of restenosis [42]. In a subsequent investigation [48,83], during the assessment of long-term outcomes of 26 AS patients, the risk factors for restenosis at short-term follow-up were the same as those seen in our first cohort [42]. Moreover, this study [48,83] suggested that the larger the number of risk factors, the greater the probability for re-obstruction (Figure 55).

Based on the information reviewed above [42,48,63], it was concluded that the patient’s age ≤ 3 years at the time of BAV and immediate post-BAV peak aortic valve gradient ≥ 30 mmHg are predictive of restenosis of aortic valve. It is further speculated that bypassing or decreasing risk factors may avoid or decrease the recurrence rate after BAV. Since the immediate post-BAV aortic valve gradients ≥ 30 mmHg is an alterable risk factor, we support utilizing balloons large enough to decrease the peak-to-peak systolic gradient to <30 mmHg [42,48,63].

#### 5.2.2. Feasibility of Repeat BAV to Address Recurrence of AS

**Figure 56:**
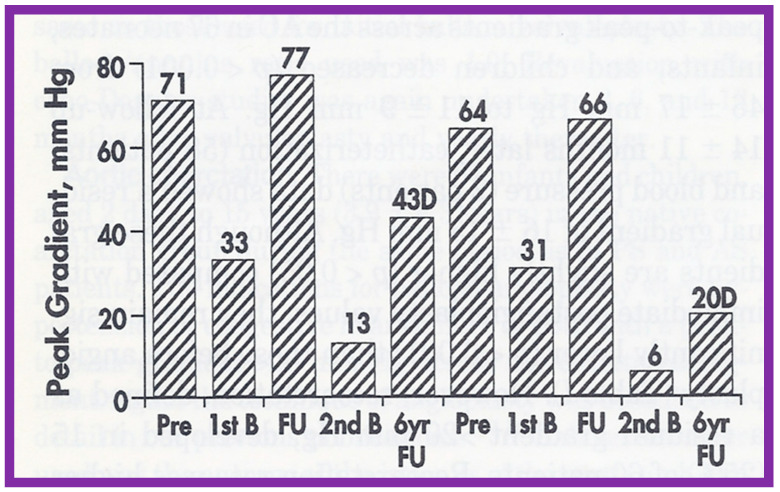
Bar graph showing aortic valve peak-to-peak systolic pressure gradients before (Pre), after initial balloon valvuloplasty (1st B), at follow-up (FU), after repeated balloon dilatation (2nd B), and at late follow-up at 6 and 7 years, respectively, in 2 patients with restenosis. Note a significant decrease in gradient after each balloon valvuloplasty. Gradients remained low after the second balloon valvuloplasty by Doppler (D) and at late follow-ups 6 and 7 years later. Reproduced from Reference [144].

As reviewed above, the recurrence of aortic stenosis after BAV was documented. The feasibility and effectiveness of repeating BAV to relieve recurrent AS were assessed in the past [144]. Twenty-six patients with AS had BAV between 1983 and 1993; peak aortic valve gradients were reduced (71 ± 20 mmHg vs. 26 ± 12 mmHg; *p* < 0.001) at the time of initial BAV. At short-term follow-up of 10 ± 4 months after BAV, residual aortic valve gradients were 34 ± 20 mmHg continued to be lower (*p* < 0.001) than pre-BAV pressure gradients but are similar in comparison with immediate post-BAV peak values. When the data on each patient was scrutinized, six (23%) of the 26 were found to have restenosis, defined as residual aortic valve pressure gradients > 50 mmHg. Four of these children had effective relief of obstruction by surgical aortic valvotomy and two children had repeat BAV. The second BAV decreased peak gradients from 77 and 66 mmHg to 13 and 6 mmHg, respectively [145] (Figure 56). Two other patients developed restenosis during long-term follow-up evaluation and the second BAV at 70 and 107 months after the initial BAV successfully relieved the obstruction. The size of the balloons used in these 4 patients is slightly larger than that used during the first BAV.

Consequently, it may be concluded that repeat BAV is feasible and effective in addressing aortic valve restenosis following a previous BAV. Therefore, we suggest that repeat BAV as the treatment of option for such children [48,83,84,144].

### 5.3. Aortic Coarctation–Native

**Figure 57:**
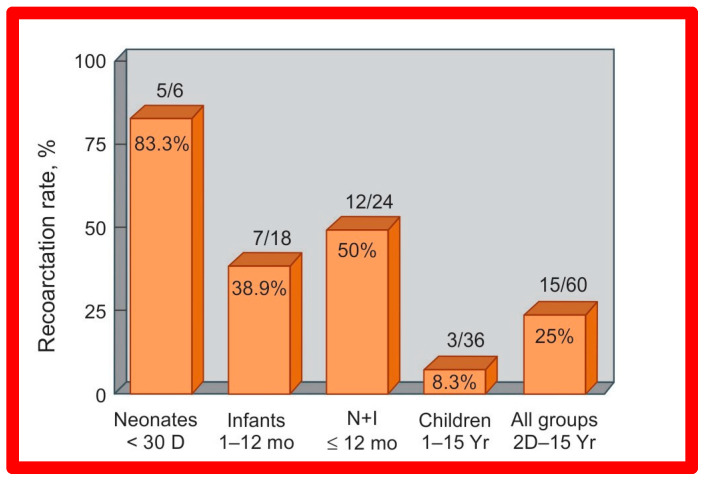
Bar graph illustrates prevalence of re-coarctation following balloon angioplasty. Percent prevalence is marked within the bar while the actual numbers are shown on the top of the bar. The prevalence is low at 8.3% in children, while it is high (83%) in neonates. D, days; I, infants; mo, months; N, neonates; Yr, years. Reproduced from Reference [94].

Short-term (14 ± 11 months) follow-up outcomes of 60 patients were evaluated by catheterization/angiography in 58 patients and by clinical evaluation in two [46,47,64]. The residual peak coarctation gradients were low at 16 ± 15 mmHg (Figure 25). The residual pressure gradients are lower (*p* < 0.001) than gradients found prior to BA (46 ± 17 mmHg) but are slightly higher (*p* < 0.05) than the gradients (11 ± 9 mmHg) seen immediately after BA (Figure 25). An example of pressure gradient recording at one-year follow-up is shown in Figure 24. Angiograms stayed improved (Figure 26 and Figure 32). The corrected aortic segment, quantified by angiograms stayed wide open. The aneurysmal formation was noticed in 5% of patients (3 of 58 patients who had follow-up angiograms). Re-coarctation, defined as peak AC gradient ≥ 20 mmHg occurred in 25% (15 of 60) patients. The younger the child at BA, the higher the chance for re-coarctation (Figure 57). The aorta became more uniform in its diameter, i.e., remodeling of the aorta took place in patients who had good results [145]. The short-term outcomes of BA of native AC reported by other investigators were reviewed elsewhere [27,39,47,64,94,138] for the interested reader. Repeat BA was performed in some patients and will be reviewed here-under [47,144].

#### 5.3.1. Causes of Restenosis

**Figure 58:**
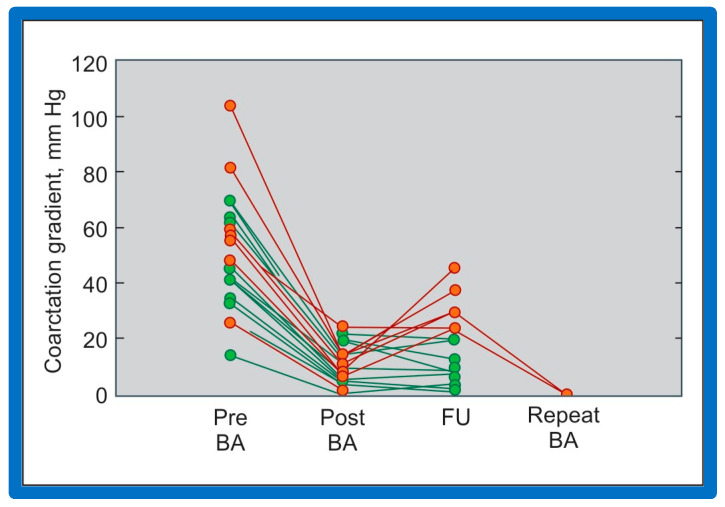
Line graph shows coarctation gradients prior to (Pre), immediately following (Post) and at follow-up (FU) after balloon angioplasty (BA). Patients with good results are shown in green while those with poor results are shown in orange. Repeat BA was performed in some patients and the gradients fell. Reproduced from Reference [94].

As discussed in the preceding section, pressure gradients across AC remain lower than those prior to BA; however, when each patient’s result is assessed individually, some children were found to have re-coarctation, defined as a peak coarctation gradient ≥ 20 mmHg with or without angiographic constriction (Figure 58).

**Figure 59:**
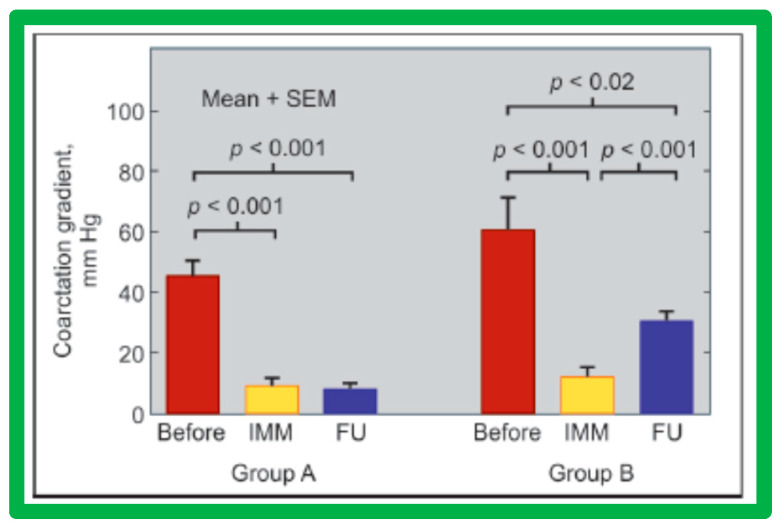
Bar graph showing immediate (IMM) and follow-up (FU) results of balloon angioplasty in Group A with good results (left panel) and in Group B with poor results (right panel). In Group A with good results, the coarctation gradients decreased significantly (*p* < 0.001) immediately after balloon angioplasty and remained low (*p* < 0.001) at follow-up. In Group B with poor results, the coarctation gradient also fell (*p* < 0.001) immediately after angioplasty but increased significantly (*p* < 0.001) at follow-up, SEM, standard error of mean. Reproduced from Reference [94].

The causes of re-coarctation after BA of native AC were investigated [146,147] in a manner like that used for restenosis of pulmonary and aortic valves [42,142]. Information on 30 patients between the ages of 14 days to 13 years who had BA was assessed. The follow-up catheterization and angiographic data in 20 children at 6 to 30 months after BA were separated into two groups: Group A. Thirteen children with good results, defined as coarctation pressure gradients ≤ 20 mmHg and no evidence for re-coarctation on angiography and Group B. Seven children with fair or poor results, defined as coarctation gradients > 21 mmHg with or without angiographic re-coarctation. In Group A patients, there was a significant reduction of coarctation gradients (*p* < 0.001) following BA and remained low (*p* < 0.001) at follow-up (Figure 59, left panel). In Group B patients, there was also a significant reduction of coarctation gradients (*p* < 0.001) immediately following BA, but, at follow-up, the pressure gradients increased significantly (*p* < 0.001) (Figure 59, right panel).

**Figure 60:**
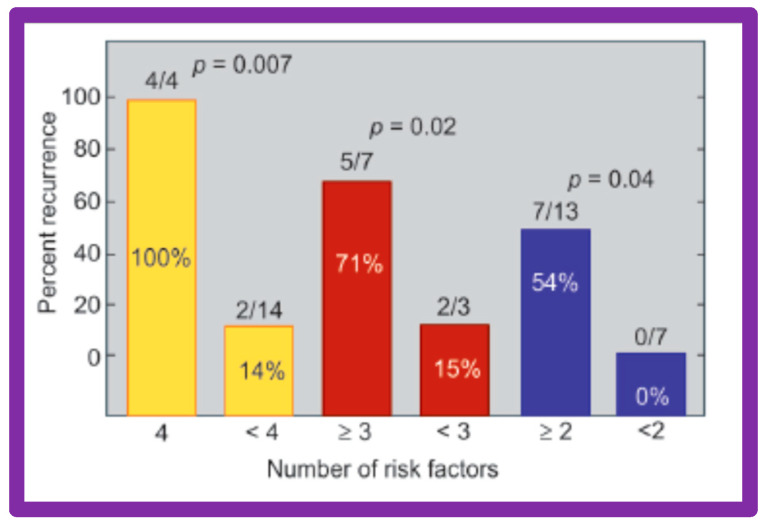
The bar graph demonstrates the influence of multiple risk factors on rates of recurrence of coarctation after balloon angioplasty. Note that the larger the number of risk factors, the greater is the probability for re-coarctation. Percentages are marked within the bars and actual numbers are shown on the top of each bar. Reproduced from Reference [94].

Thirty general, local anatomical, physiological, and technical data [146,147] were analyzed by multivariate logistic regression testing. This analysis identified four issues as risk factors for the recurrence of coarctation. These are as follows: 1. Age less than 12 months at the time of BA, 2. Aortic isthmus less than two-thirds the size of the ascending aorta immediately proximal to the right innominate artery, 3. Coarcted aortic segment < 3.5 mm prior to BA, and 4. Coarcted aortic segment < 6 mm immediately following BA. In addition, it was observed that the existence of two or more risk factors is linked with a higher rate of re-coarctation; the greater the number of risk factors, the higher the likelihood for re-coarctation (Figure 60).

The detection of risk factors is expected to assist in the selection of patients for BA. Circumventing or reducing the number of risk factors may lower the prevalence of the re-coarctation rate after BA. The above-described data were revalidated when the data on a larger number of patients (N = 58) was utilized [47,148].

#### 5.3.2. Feasibility of Repeat BA to Address Recurrence of AC

**Figure 61:**
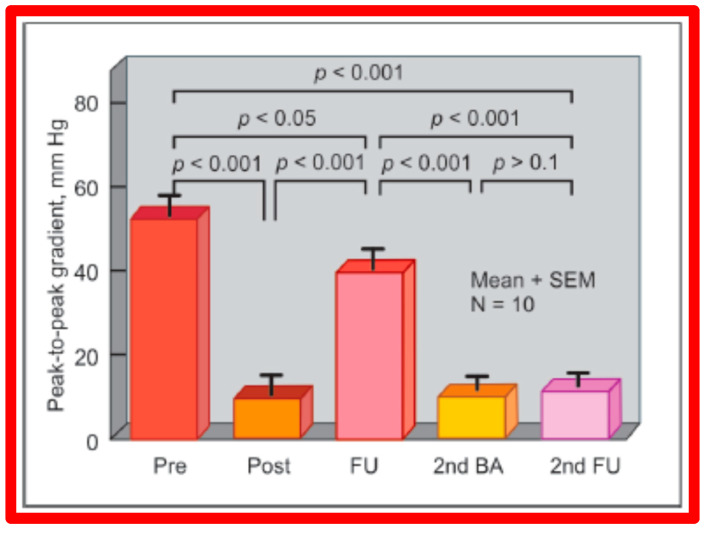
Results of repeat balloon angioplasty of aortic coarctation that had restenosis after initial balloon angioplasty. Initial gradients were reduced (*p* < 0.001) significantly after angioplasty (pre vs. post), which returned toward pre-angioplasty values (*p* < 0.05) at intermediate-term follow-up (FU). Repeat balloon angioplasty (2nd BA) again reduced the gradient (*p* < 0.001) which remained similar (*p* > 0.1) on further follow-up (2nd FU). The residual gradients continue to be lower than the gradients prior to the first (*p* < 0.001) and second (*p* < 0.001) balloon procedures. Mean + standard error of mean (SEM) is shown. N = 10 (number of subjects undergoing repeat balloon angioplasty). Reproduced from Reference [94].

As reviewed in the preceding section, re-coarctation after BA of AC occurs. We have evaluated the feasibility and effectiveness of repeat BA in relieving restenosis [144]. A total of sixteen patients developed re-coarctation; twelve of these patients had repeat BA. At the time of initial BA, peak AC pressure gradients were reduced significantly (49 ± 17 vs. 10 ± 9 mmHg; *p* < 0.001) but risen to 38 ± 11 mmHg; *p* < 0.001) at follow-up (Figure 61). Repeat BA was accomplished in these children with catheters carrying balloons slightly bigger than those utilized at the time of initial BA (but no bigger than the size of the descending aorta at the diaphragm). A decrease (38 ± 11 mmHg vs. 10 ± 6 mmHg; *p* < 0.001) of peak coarctation gradients took place at the time of the second BA (Figure 61). Follow-up 25 ± 15 months later, the arm-to-leg pressure gradient, measured by blood pressures (BP) stayed low (11 ± 6 mmHg) and largely unchanged (*p* > 0.1) from those of second post-BA values (Figure 61). Additional follow-up at a mean of 5 years, the arm-to-leg BP gradients continued to be low (9 ± 9 mmHg).

In another study of AC in neonates and infants less than 3 months of age, conducted by the author [98], 14 infants with post-BA re-coarctations underwent repeat BA with resultant reduction in peak systolic pressure gradients (54 ± 20 mmHg vs. 9 ± 7 mmHg; *p* < 0.001). At a median follow-up of three years, the arm-to-leg BP difference was low at 4 ± 6 mmHg. These data would also indicate the feasibility and effectiveness of repeat BA to successfully address post-BA re-coarctations.

Based on the data from both the above studies, we concluded that repeat BA is feasible and effective in alleviating re-obstruction that formed following the initial BA of AC [98,144].

### 5.4. Aortic Coarctation, Postsurgical

**Figure 62:**
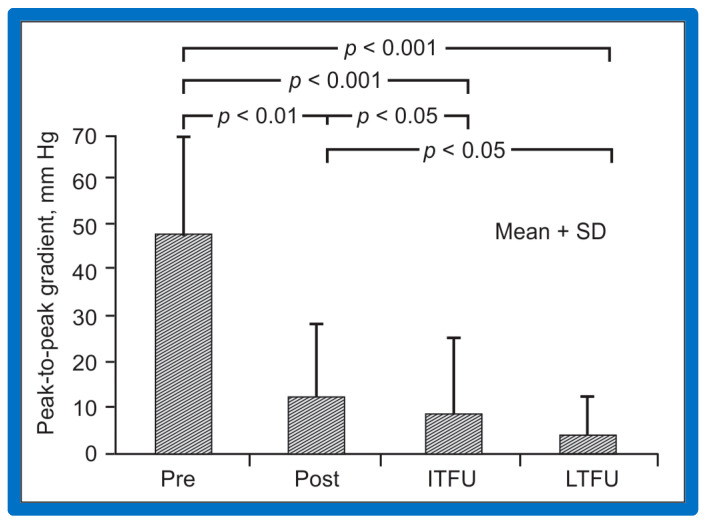
Bar graph of immediate and follow-up results after balloon angioplasty of postsurgical aortic re-coarctation are shown. Peak-to-peak systolic pressure gradients across the coarctation decreased significantly (*p* < 0.001) from prior to (Pre) to immediately after (Post) balloon angioplasty. They decreased further (*p* < 0.05) at intermediate-term (ITFU) and at long-term follow-up (LTFU). The ITFU and LTFU gradients remained remarkably lower (*p* < 0.001) than those of prior balloon angioplasty. Mean + SD (standard deviation) is shown. Reproduced from Reference [49].

**Figure 63:**
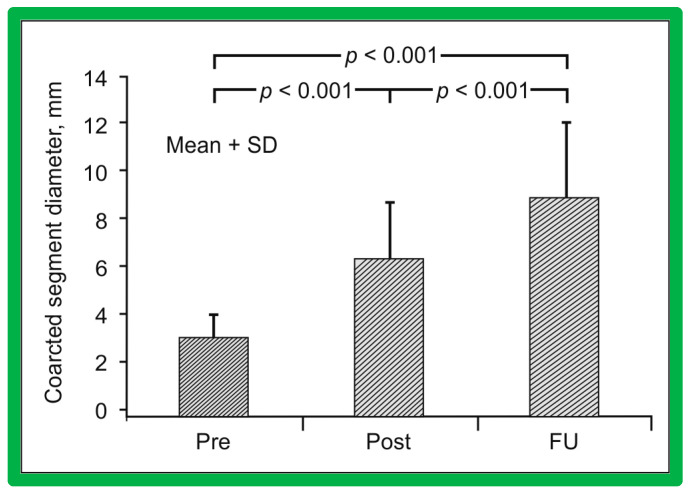
A bar graph of immediate and follow-up results after balloon angioplasty of postsurgical aortic re-coarctation are shown. Coarctation segment diameters increased significantly (*p* < 0.001) from prior to (Pre) or immediately after (Post) balloon angioplasty. They increased further (*p* < 0.001) at intermediate-term follow-up (FU). The FU coarctation segment diameters remained wider (*p* < 0.001) than those of prior balloon angioplasty. Mean + SD (standard deviation) is shown. Reproduced from Reference [49].

Short-term follow-up outcomes from our study group of postsurgical re-coarctations [49] showed a sustained reduction in the peak re-coarctation systolic pressure gradient (9 ± 16 mmHg; *p* < 0.05) (Figure 62) and improvement in the diameter of the dilated coarctation segment (6.4 ± 2.4 mm vs. 9 ± 3 mm; *p* < 0.001) (Figure 63). Figure 34, Figure 35, and Figure 39 illustrate angiographic improvement at short-term follow-up. The findings described by other investigators were charted in our previous papers [45,49,65,94] and are comparable to those of ours [49]. We also noted improvement in the diameter of the transverse aortic arch/aortic isthmus from 7 ± 3 to 10 ± 3 (*p* < 0.01) at short-term follow-up, suggesting remodeling of the aorta such as that shown following successful BA of native AC [146].

While there are no re-coarctations in our study subjects [49], the presentation of follow-up results described in the literature at the time of our review [49] showed the development of restenosis, defined as peak gradient ≥ 20 mmHg in 18% of the patients. Likewise, aneurysms were not seen at follow-up angiographic and/or MRI studies from our study subjects [49]; however, studies from other investigators revealed aneurysms in 6% (9 out of 142) patients; these were tabulated/reviewed in our prior publications [49,94] for the interested reader.

Since there is no evidence for re-coarctations in our study cohort, a discussion of the causes of re-coarctation and the feasibility of re-dilatation is not necessary.

## 6. Long-Term Results

Long-term, defined as greater than five years following balloon dilatation, will be examined for each of the lesions under discussion (PS, AS, and AC).

### 6.1. Pulmonary Stenosis

The outcomes of 80 patients with PS who had BPV at a follow-up duration of a median of seven years (three to ten years) were reviewed [50]; these findings will be examined under the following headlines.

#### 6.1.1. Residual Gradients

**Figure 64:**
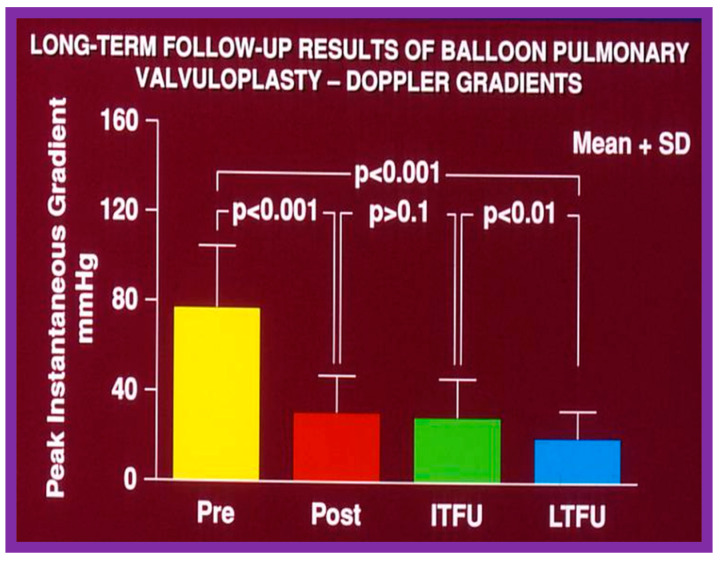
Bar graph demonstrating maximum peak instantaneous Doppler gradients prior to (Pre) and one day following (Post) balloon pulmonary valvuloplasty, and at intermediate-term (ITFU) and long-term (LTFU) follow-up. Note the significant reduction (*p* < 0.001) after valvuloplasty, which remains unchanged (*p* > 0.1) at ITFU. However, at LTFU there was a further fall (*p* < 0.001) in the Doppler gradients. The mean + standard deviation (SD) is shown. Modified from Reference [50].

The long-term follow-up residual peak instantaneous Doppler gradients across the pulmonary valve were extremely low at 17 ± 12 mmHg; these pressure gradients were lower than the pre-BPV gradients (*p* < 0.001) and the pressure gradients recorded both at immediate post-BPV and short-term follow-up values (*p* < 0.001) (Figure 64) [50].

#### 6.1.2. Ventricular Dimensions

**Figure 65:**
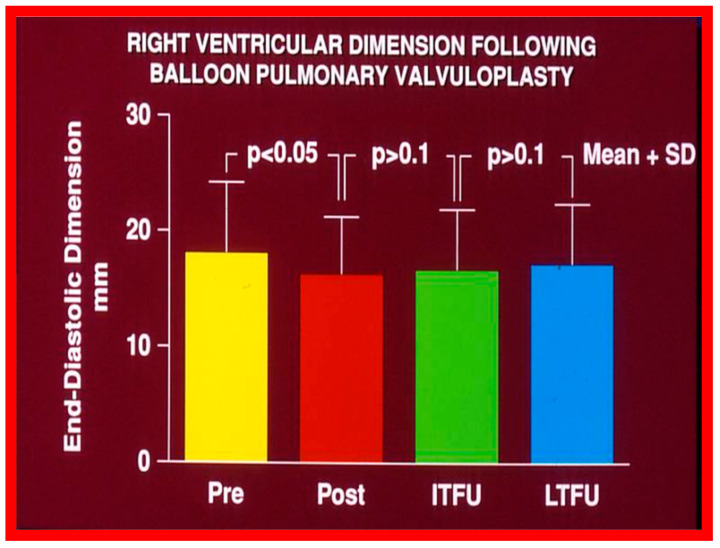
Bar graph showing the right ventricular end-diastolic dimensions prior to (Pre) and one day after (Post) balloon pulmonary valvuloplasty, and at intermediate-term (ITFU) and at long-term (LTFU) follow-up. There was a significant decrease (*p* < 0.05) in right ventricular size immediately following the balloon procedure. There was no further change at ITFU and LTFU. A significant increase (*p* < 0.05) in the incidence of flat septal motion was observed at LTFU (see Figure 10, Figure 11, Figure 12, Figure 13, Figure 14, Figure 15, Figure 16, Figure 17, Figure 18 and Figure 19). No patient had paradoxical septal motion. The mean + standard deviation (SD) is shown. Modified from Reference [50].

Even though there was a substantial reduction (*p* < 0.05) in the RV end-diastolic dimension at the time of BPV, there was no additional change (*p* > 0.1) both at short- and long-term follow-up evaluation (Figure 65) [50]. Furthermore, the RV dimensions were within the normal limits for the square root of the body surface area for a given age. The LV end-diastolic dimensions increased (*p* < 0.01) at long-term follow-up evaluation when compared with the pre-BPV measurements; this was believed to be due to the growth of the LV, proportionate to the increasing patient’s age. Additionally, the LV dimension was within the normal limits for the square root of the body surface area [50].

#### 6.1.3. Re-Interventions and Actuarial Event-Free Rates

**Figure 66:**
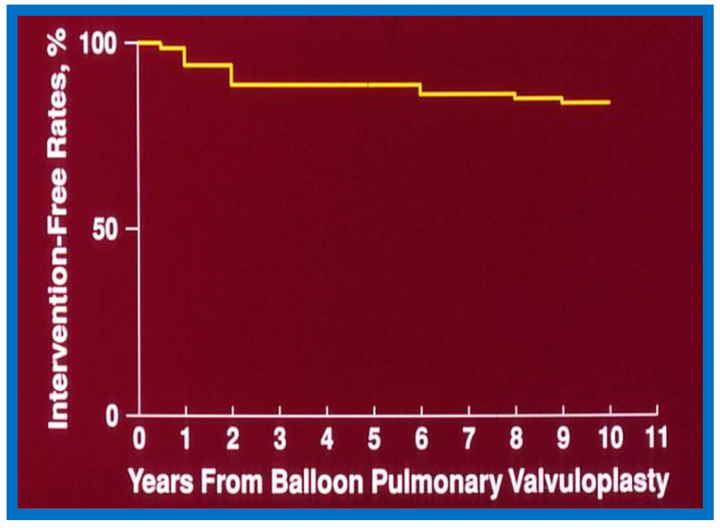
Actuarial event-free rates after balloon pulmonary valvuloplasty. The re-intervention-free rates at one, two, five, and ten years after the procedure are 94%, 89%, 88%, and 84%, respectively. Modified from Reference [50].

No patient from our study subjects required re-intervention immediately after BPV. However, seven patients required repeat BPV to address restenosis during short-term follow-ups. In addition, two other patients had resection of fixed infundibular obstructive lesions during this time. Four children had re-interventions during long-term follow-up; these late re-interventions were surgical procedures in two patients to alleviate supravalvar pulmonary artery stenosis in one and infundibular pulmonary stenosis in another and repeat BPV in two patients to address recurrent PS. The actuarial re-intervention-free rates were calculated using the Kaplan–Meir technique [149]. The actuarial re-intervention-free rates at five and ten years were in the high and mid-80s, respectively (Figure 66) [50].

#### 6.1.4. Development of Pulmonary Insufficiency

**Figure 67:**
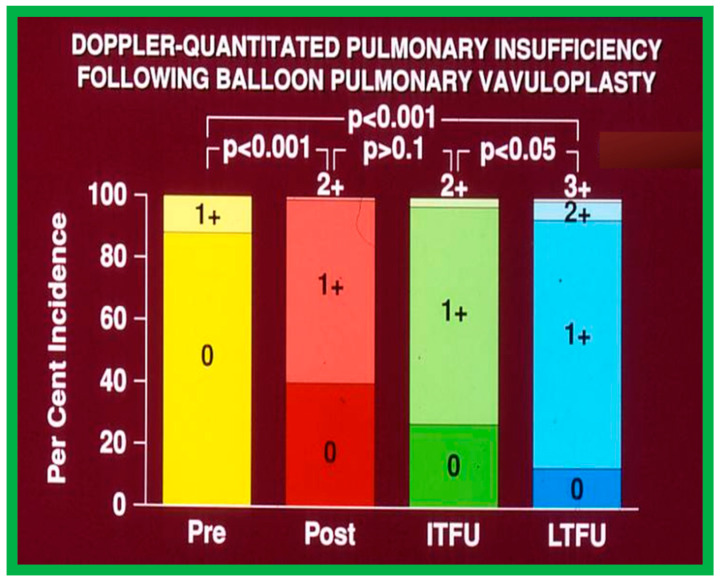
Bar graph showing Doppler graded pulmonary insufficiency (PI) prior to (Pre) and one day after (Post) balloon pulmonary valvuloplasty and at intermediate-term (ITFU) and long-term (LTFU) follow-up. 0, No PI; 1+, 2+, 3+, PI grade as per table above. A gradual but significant increase (*p* < 0.05 to *p* < 0.001) in the incidence of PI is seen. Modified from Reference [50]. Dark color indicates less degree of PI and light color indicates more severe degree of PI.

**Figure 68:**
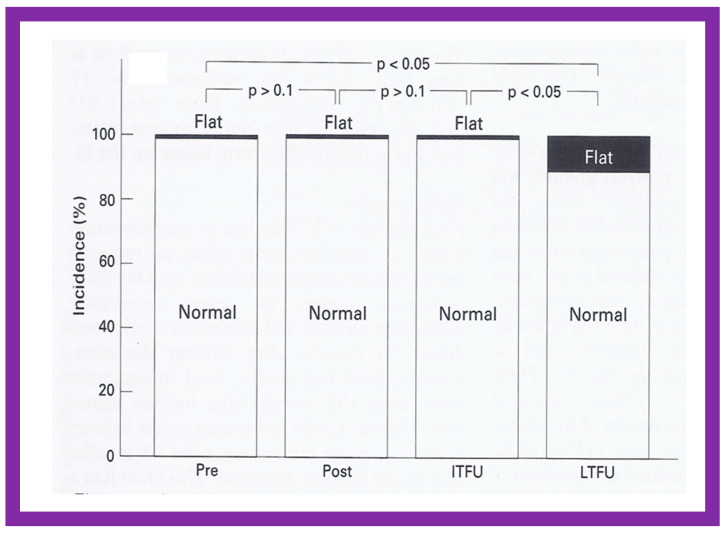
Bar graph showing the prevalence of inter-ventricular septal motion prior to (Pre) and one day after (Post) balloon pulmonary valvuloplasty, and at intermediate-term (ITFU) and long-term (LTFU) follow-up. Note the significant increase (*p* < 0.05) in the incidence of flat septal motion at LTFU. No patient was observed to have paradoxical septal motion. Reproduced from Reference [50].

A grading system was designed to estimate the extent of PI as shown in Table 1 of [50]. A gradual but substantial (*p* < 0.05 to *p* < 0.001) increase in the frequency of pulmonary insufficiency (PI) was seen from pre-BPV to one day after BPV, and then during short-term and long-term follow-up evaluation (Figure 67). The frequency of occurrence of flat inter-ventricular septal motion was also seen (Figure 68), which may suggest that RV volume overload is developing due to PI, but no patient developed paradoxical inter-ventricular septal motion.

#### 6.1.5. Summary of Long-Term Results

In short, the long-term outcomes of BPV are excellent, with an occasional requirement for re-intervention. These data assert that BPV is the therapy of choice in the management of valvar PS; nevertheless, our study points out concern regarding the development of PI [50] at long-term follow-up. The long-term results reported by other investigators were reviewed elsewhere [50,62,75] for the interested reader.

### 6.2. Aortic Stenosis

We examined long-term follow-up outcomes of 26 children who were reevaluated 3 to 10 years (6.7 ± 1.7 years) after BAV. Twenty-two of these children were restudied longer than 5 years following BAV [48]. These data will be reviewed under the following headlines:

#### 6.2.1. Residual Gradients

Residual stenosis, as assessed by peak instantaneous Doppler gradients, at long-term follow-up was minimal with a gradient of 27 ± 17 mmHg (Figure 21). These gradients are lower than pre-BAV values (*p* < 0.001) but are comparable (*p* > 0.1) to both immediate post-BAV and short-term follow-up gradients (Figure 21) [48].

#### 6.2.2. Ventricular Dimensions and Function

The LV end-diastolic dimension (45.4 ± 9.9 mm) at long-term follow-up evaluation, was larger (*p* < 0.01) when compared with both immediate post-BAV (37.2 ± 0.5 mm) and pre-BAV (36.7 ± 8.5 mm) values (Figure 23, left panel). To circumvent the potential influence of growth, LV measurements were standardized to the square root of the body surface area. The resulting measurements were: 38.5 ± 42 vs. 49.9 ± 5.7 mm/m^2^ (*p* < 0.001); thus, the data continue to demonstrate that the LV end-diastolic diameter is larger at long-term follow-up, presumably related to the undesirable effect of AI. However, there was no significant change (*p* > 0.05) in the LV posterior wall thickness in diastole (8.3 ± 1.7 mm) (Figure 23, middle panel) and LV fractional shortening (45 ± 6%) (Figure 23, right panel) at long-term follow-up.

#### 6.2.3. Re-Interventions and Actuarial Event-Free Rates

**Figure 69:**
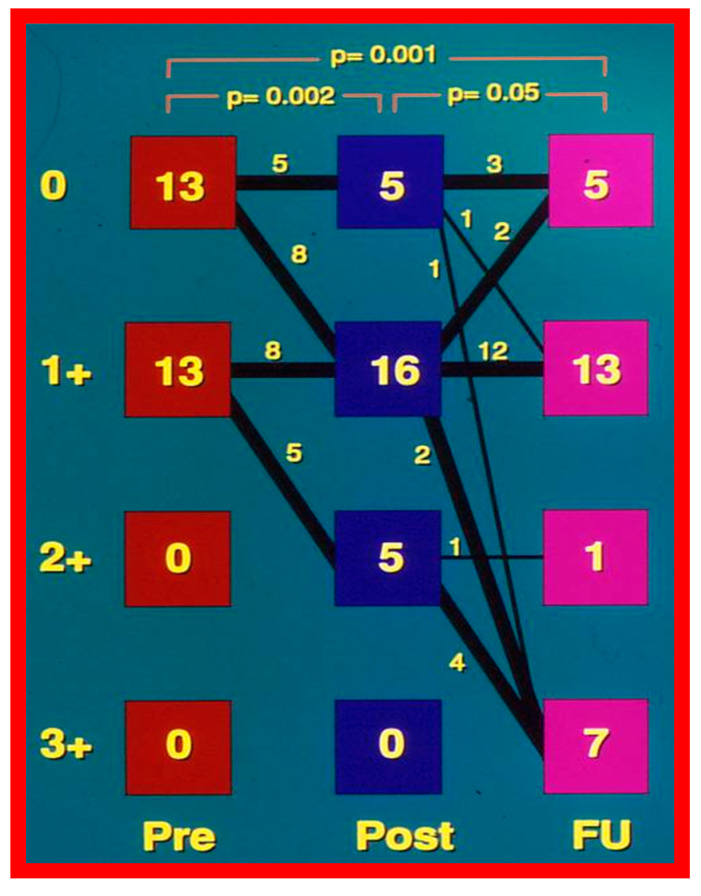
Degree of aortic insufficiency by Doppler echocardiography before (Pre), the day after (Post), and at late follow-up (FU). There is a significant (*p* = 0.002) increase in aortic insufficiency from pre-valvuloplasty to post-valvuloplasty. The number of patients with grade 3+ aortic insufficiency (0 of 26 vs. 7 of 26) at follow-up (FU) increased (*p* < 0.02). Modified from Reference [48].

A total of eight patients, 31% of the total, required re-intervention to address aortic valve restenosis; six of these occurred during short-term follow-up and two during long-term follow-up. Four patients were treated with aortic valvotomy by surgery and the other four were addressed with repeat BAV, all with successful results. In addition, a single patient required LV apex-to-descending aortic conduit to bypass severe mid-cavitary LV obstruction. While seven (27%) patients developed significant AI during long-term follow-up (Figure 22 and Figure 69) only two of these patients required Ross procedure.

**Figure 70:**
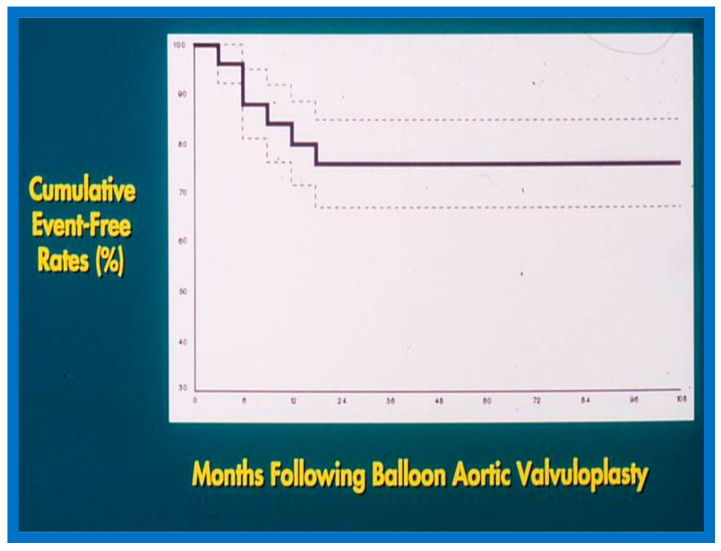
Actuarial event-free rates after balloon aortic valvuloplasty. Seventy percent confidence limits are marked with dashed lines. Note that intervention-free rates at 1, 2, 5, and 9 years are 80%, 76%, 76%, and 76%, respectively. Modified from Reference [48].

Based on these data, Kaplan–Meir even-free rates [149] were calculated. The probability of freedom from re-intervention was 80%, 76%, 76%, and 60% at 1-, 2-, 5- and 10-year follow-ups, respectively (Figure 70) [48].

#### 6.2.4. Development of Aortic Insufficiency

The amount of AI was calculated by the ratio of the color Doppler jet width of the AI to the diameter of the LV outflow as defined in our prior publication [48]. Although there was no substantial change in the magnitude of AI both at the time of BAV or at short-term follow-up (Figure 22), the number of children with 3 + AI increased at long-term follow-up (*p* < 0.01) (Figure 22 and Figure 69). Seven (28%) children had 3 + AI; in these children, the LV end-diastolic diameter was at or larger than the 90th percentile for the body surface area. Two (8%) of these children had uneventful Ross procedures. The outstanding five children were clinically followed without surgical intervention at the time of the conclusion of the study [48]. It was pointed out that AI is a highly significant long-term drawback with BAV; however, such an issue is like the long-term follow-up results of aortic valve surgery. Discussion of probable causes of AI will be undertaken in Part II of this series.

#### 6.2.5. Summary of Long-Term Results

In summary, the long-term results of BAV show sustained relief of aortic valve obstruction for the whole cohort with a hint for minimal further restenosis, continuing increase in the incidence of AI, dilatation of the left LV and somewhat high re-intervention rates [48,84,148]. Long-term follow-up results of BAV reported by other investigators are reasonably like those of ours as discussed in our prior publications [31,48,63,83,84,150].

### 6.3. Aortic Coarctation–Native

**Figure 71:**
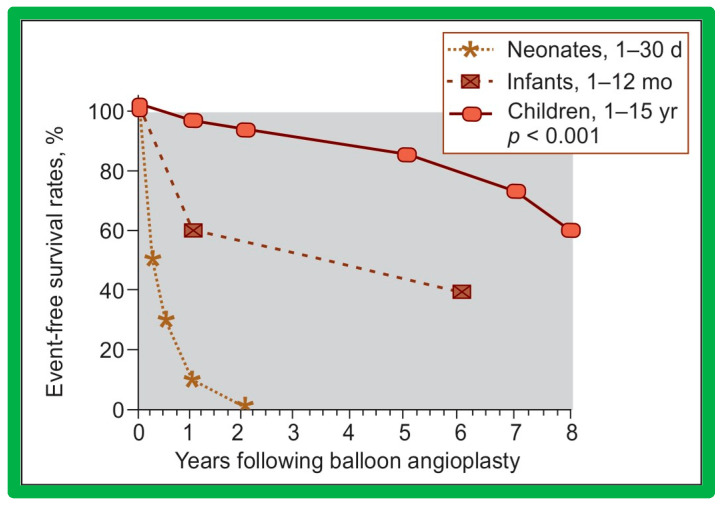
Actuarial event-free survival curves of neonates (<30 days), infants (1–12 months), and children (1–15 years) who had undergone balloon angioplasty of aortic coarctation. The event-free survival rates are better for the children group than for the neonatal and infant groups (*p* < 0.001). Modified from Reference [131].

We have analyzed long-term follow-up data of 60 BA procedures of native AC [47,51]. This analysis suggested that while re-coarctation and aneurysms manifested, some needed re-intervention during short-term follow-up, as reviewed in the preceding section, and the long-term results (5–9 years) seem reassuring. There was a minimal incidence of additional re-coarctation and no late aneurysmal formation [47,51]. In nearly all patients, the arm BP stayed normal, and the BP-determined arm/leg pressure gradient remained low (Figure 25, right-most panel). Actuarial event-free survival rates are age-dependent, the younger the age at initial BA, the higher the requirement for re-intervention (Figure 71) [47,51]. The long-term follow-up results reported by other cardiologists are essentially like those of ours, as referenced elsewhere [27,30,46,47,94,138].

### 6.4. Aortic Coarctation, Postsurgical

In the past, we have documented long-term (5 ± 2 years) follow-up outcomes of BA of post-operative aortic re-coarctation [49,51]. At long-term follow-up, the residual pressure gradients across the balloon-dilated re-coarctation site were low (5 ± 8 mmHg) (Figure 62, Right-most panel). The level of gradient reduction was independent of the types of surgical repair (end-to-end anastomosis, subclavian flap angioplasty, patch angioplasty, or repair of interrupted aortic arch) that the patients initially had [49]. However, when the results of each individual patient were examined, arm-to-leg systolic BP gradient higher than 20 mmHg occurred in two of 32 (6%) patients; these patients underwent surgery during long-term follow-up. However, it should be noted that the surgery recommendation is largely based on the presence of markedly hypoplastic aortic isthmus [49,51], not because of the re-coarctation of the balloon-dilated aortic segment.

Arm BP measured at the latest follow-up examination was 111 ± 13 mmHg; this value was significantly lesser than that recorded pre-BA (135 ± 22 mmHg; *p* < 0.001) and on the day after BA (122 ± 22 mmHg; *p* < 0.05). When compared with age-specific percentiles [151], only 3% of patients had systolic BPs higher than the 95th percentile; these were 82% prior to BA and 42% on the day after BA (*p* < 0.001).

Echo-Doppler studies were performed at the latest clinical evaluation. The LV end-diastolic dimension (35 ± 8 mm vs. 30 ± 7; *p* < 0.001) and LV posterior wall thickness in diastole (6 ± 1.6 mm vs. 5 ± 1.1 mm; *p* < 0.05) were higher at long-term follow-up than the measures secured immediately after BA. These data probably represent the growth of LV with aging rather than the failure of regression of these measures following BA since these values were within the normal range for body surface area [152]. However, the LV fractional shortening (41 ± 5% vs. 39 ± 8%; *p* = 0.9) did not alter at long-term follow-up. The peak Doppler flow velocity through the balloon-dilated coarcted segment at long-term follow-up was lower when compared to pre-BA (2.5 ± 0.6 m/s vs. 3.5 ± 0.8 m/s; (*p* < 0.01) but is similar (*p* > 0.1) to immediate post-BA measurements of 2.4 ± 0.6 m/s) (Figure 40). In a similar fashion, peak instantaneous Doppler gradients were low at long-term follow-up at 23 ± 11 mmHg which continue to lower (*p* < 0.001) than those when compared to pre-BA values (47 ± 22 mmHg) but similar (*p* > 0.1) to immediate post-BA gradients (20 ± 10 mmHg). In addition, diastolic extension of the Doppler velocity signal was not seen in any children at long-term follow-up. The long-term results reported by other investigators also indicated good outcomes as referenced elsewhere [46,49,51,94].

Thus, these data indicate excellent long-term follow-up of BA of postsurgical aortic re-coarctations.

## 7. Epilogue

Several other observations have been made during our studies of balloon dilatation procedures of PS, AS, and AC. Due to the limitations of space, these observations/phenomena/innovations will be reviewed in Part II of this series.

## 8. Summary and Conclusions

Percutaneous vascular dilatation techniques described by Dotter, Grüntzig, and their associates were adopted to relieve congenital obstructive lesions of the heart. This review describes indications, techniques, and outcomes of balloon dilatation in patients with PS, AS, and AC. In patients with PS, BPV results in immediate reduction of peak pressure gradients across the pulmonary valve which was maintained at short- and long-term follow-up in most patients. Recurrent PS occurred in a small percentage of patients and was addressed successfully by repeat BPV. BPV has become the choice procedure in the management of valvar PS. However, reports of the occurrence of pulmonary insufficiency at long-term follow-up is of concern. In patients with AS, BAV produces an immediate decline in peak pressure gradients across the aortic valve which stayed low at short- and long-term follow-up in most children. Recurrent AS occurred in some patients which were treated effectively by repeat BAV. BAV has become the first option in the management of valvar AS. Nevertheless, the appearance of aortic insufficiency at long-term follow-up is troublesome. BA of both native and postsurgical AC is effective with a reduction in pressure gradients across the AC and improvement in CHF and hypertension. The effectiveness persisted at both short- and long-term follow-ups in most patients. Recurrent AC happened in several patients which were treated effectively by repeat BA or surgery. The occurrence of aneurysms, though rare, is of concern. The method of management of native AC is controversial; some institutions prefer surgery and others BA. However, BA is the treatment of choice for addressing post-operative recurrent AC. The author recommends that future studies should focus on devising methods to prevent valvar insufficiency in PS and AS cases and reduce/prevent aneurysm formation in AC patients.

## Data Availability

Not applicable.
